# The role of KDEL-tailed cysteine endopeptidases of *Arabidopsis* (AtCEP2 and AtCEP1) in root development

**DOI:** 10.1371/journal.pone.0209407

**Published:** 2018-12-21

**Authors:** Timo Höwing, Marcel Dann, Benedikt Müller, Michael Helm, Sebastian Scholz, Kay Schneitz, Ulrich Z. Hammes, Christine Gietl

**Affiliations:** 1 Lehrstuhl für Botanik, Center of Life and Food Sciences Weihenstephan, Technische Universitaet Muenchen, Freising, Germany; 2 Cell Biology and Plant Biochemistry, Biochemie-Zentrum Regensburg, University of Regensburg, Regensburg, Germany; 3 Plant Developmental Biology, Center of Life and Food Sciences Weihenstephan, Technische Universitaet Muenchen, Freising, Germany; Hainan University, CHINA

## Abstract

Plants encode a unique group of papain-type cysteine endopeptidases (CysEP) characterized by a C-terminal KDEL endoplasmic reticulum retention signal (KDEL-CysEP) and an unusually broad substrate specificity. The three *Arabidopsis* KDEL-CysEPs (*AtCEP1*, *AtCEP2*, and *AtCEP3*) are differentially expressed in vegetative and generative tissues undergoing programmed cell death (PCD). While KDEL-CysEPs have been shown to be implicated in the collapse of tissues during PCD, roles of these peptidases in processes other than PCD are unknown. Using mCherry-AtCEP2 and EGFP-AtCEP1 reporter proteins in wild type versus *atcep2* or *atcep1* mutant plants, we explored the participation of AtCEP in young root development. Loss of AtCEP2, but not AtCEP1 resulted in shorter primary roots due to a decrease in cell length in the lateral root (LR) cap, and impairs extension of primary root epidermis cells such as trichoblasts in the elongation zone. AtCEP2 was localized to root cap corpses adherent to epidermal cells in the rapid elongation zone. *AtCEP1* and *AtCEP2* are expressed in root epidermis cells that are separated for LR emergence. Loss of *AtCEP1* or *AtCEP2* caused delayed emergence of LR primordia. KDEL-CysEPs might be involved in developmental tissue remodeling by supporting cell wall elongation and cell separation.

## Introduction

Plants encode a unique group of papain-type cysteine endopeptidases (CysEP) characterized by a C-terminal KDEL endoplasmic reticulum (ER) retention signal (KDEL-CysEP) with RcCysEP from castor bean (*Ricinus communis*) as the founding member [1–3]. KDEL-CysEPs are not found in mammals or fungi, but are ubiquitous in spermatophytes; the number of homologous KDEL-CysEP genes present in seed plant genomes is variable and indicates several clade specific duplication events [4].

KDEL-CysEPs are synthesized as pre-pro-enzymes and are co-translationally transferred into the ER, where the pre-sequence signal peptide is removed. KDEL-CysEPs are stored as enzymatically inactive pro-proteins in ER-derived compartments known as ricinosomes, KDEL-tailed protease-accumulating vesicles (KVs) and ER-3bodies [2, 3, 5–7]. Upon acidification, the KDEL-CysEPs are released; the pro-sequence together with the C-terminal KDEL ER retention signal are removed resulting in activation of the enzyme [3, 6, 8] The mature KDEL-CysEPs exhibit unusually broad substrate specificity and digest cytoplasmic proteins in tissues that collapse during the final stages of programmed cell death (PCD), allowing for transfer of the cleavage products to the surviving parts of the plant [9]. In addition, they are unique in being able to digest extensins [10], which form the basic scaffold of the cell wall [11]. The broad substrate specificity is due to the active site cleft of the KDEL-CysEPs, which accepts a wide variety of amino acids including proline and the glycosylated hydroxyproline of the hydroxyproline rich glycoproteins of the cell wall [12]. The amino acid residues that are important for this generally more open structure of the active site cleft, together with those defining the catalytic pocket, are highly conserved among the known KDEL-CysEPs [4].

KDEL-CysEPs, together with other classes of proteases including serine proteases, aspartic proteases and metalloproteases, have well defined roles during PCD [13–15]. In plants, PCD occurs during development and senescence, under stress conditions, and in response to pathogen infection. In plant-microbe pathogenic interactions, PCD must be tightly controlled. Plants limit the spread of pathogens by rapid cell death at the site of infection through a mechanism known as the hypersensitive response (HR; [16]). The growth of biotrophic pathogens is restricted by PCD because these depend on living host tissue to feed on. KDEL-CysEPs participate in developmental PCD, particularly for the elimination of tissues and cells serving transient functions during development, such as megagametophyte cells after germination of white spruce (*Picea glauca*) seeds [17], senescing endosperm tissue of germinating and collapsing nucellus cells of maturing castor bean seeds [2, 3, 9], endosperm cells of imbibed tomato seeds [18], tapetum cells in anthers [19, 20] or *Lilium longiflorum* tepals [21], the inner integument from developing seeds of *Jatropha curcas* [22]. Together with nucleases and other proteases, KDEL-CysEPs play a fundamental role in PCD during development (for recent reviews see [23, 24]). While the role of KDEL-CysEPs in PCD has been extensively characterized, whether these proteases have roles in processes other than PCD remains unclear.

In *Arabidopsis*, three KDEL-CysEPs—AtCEP1 (At5g50260), AtCEP2 (At3g48340), and AtCEP3 (At3g48350)—have been identified and shown to act in a tissue- and organ-specific fashion during seedling, flower, and root development [6, 10, 25]. CEP1 is a factor of basal resistance to powdery mildew caused by the biotrophic ascomycete *Erysiphe cruciferarum*, and is expressed in spatiotemporal association with the late fungal development on *Arabidopsis* leaves [26, 27]. *CEP1* (together with *METACASPASE9*, *BFN1* and *PASPA3*) is also associated with or functionally implicated in developmental PCD in several *Arabidopsis* cell types [28]. *CEP2* expression has been detected in the epidermal layers of leaves, hypocotyls and roots, especially in the root cap cells and at the upper end of the lateral root (LR) cap (PCD site I), as well as during LR emergence [6, 10], but the role in root development had, to date, not been elucidated. Interestingly, *Arabidopsis* KDEL-CysEPs are expressed not only in tissues undergoing PCD, but also in tissues not known to undergo PCD [6, 10].

The aim of this study was to explore the participation of CEPs in processes other than PCD. Root development was used as a model system for cell elongation and cell separation in young seedlings.

## Materials and methods

### *Arabidopsis* mutant plants

Homozygous ko mutant plants were obtained for *cep1* (SAIL_158_B06, [26]) and for *cep2* (SALK_079519; T-DNA insertion in the second exon) by segregation analysis and genotyping. We performed three reciprocal back crossings in order to remove T-DNA insertions elsewhere in the genome. Transcription analysis confirmed homozygous *cep1* ko [26] and *cep2* ko mutant plants (S1 Fig). During back-crossing of the *cep2* mutant allele, we recovered homozygous *cep2* ko mutant plants. However, even by consecutive back crossing we were not able to recover Mendelian segregation of the *cep2* mutant and WT alleles: No homozygous WT plants resulted from the back crosses, indicating a secondary T-DNA insertion which could not be removed. We refrained therefore from using the *cep2* mutant allele for further crosses and modifications such as *cep1 cep2* double mutant generation or transformation with reporter constructs. Since no second *cep2* insertion line was available, we used two independent *cep2*-RNAi lines in order to confirm the observed *cep2* ko mutant phenotype. *cep1* mutant plants behaved like WT in the context of our research concerning primary root elongation (see Results). We used the *cep2*-RNAi construct to transform *cep1* mutant plants in order to analyze *cep2* knock down (kd) mutants in the *cep1* background. Silencing of *CEP2* was achieved using pHANNIBAL and the binary vector pART27 to accept the NotI fragment from pHANNIBAL (CSIRO Plant Industry, Canberra ACT 2601, Australia), and the *Agrobacterium tumefaciens* strain GV3010::pMP90. A representative *CEP2* region in the 3’UTR comprising 134 bp was amplified from TAMU-BAC T29H11 as BamHI/XhoI-fragment and as ClaI/KpnI-fragment, respectively, using the primers 5’-AAC GGA TCC TCG AGA GAC TTT AAG TCA TTG AAA ACT G-3’ and 5’-TGG ATC GAT GGT ACC TGC TGG CCA ATA TTA CAA GGA G-3’. The resulting PCR products were cloned into pHANNIBAL as BamHI-ClaI-fragment and as XhoI-KpnI-fragment, and the final plasmid was sequenced. The NotI-fragment was cloned into pART27, and the final plasmid was transformed into *A*. *tumefaciens* by electroporation. Flowers from homozygous *cep1* ko mutant plants were transformed by floral dipping [29] with *A*. *tumefaciens* harboring the *CEP2*-silencing plasmid and in parallel with *A*. *tumefaciens* harboring the *CEP2*-reporter including mCherry without the mature *CEP2* protein (P_CEP2_::pre-pro-3xHA-mCherry-KDEL, [6]) resulting in plants silencing *CEP2*. Primers used for qRT-PCR were: ACT8 qRT forward: TGAGACCTTTAATTCTCCAGCTATG; ACT8 qRT reverse: CCAGAGTCCAACACAATACCG; CEP2 qRT forward: GCTGTTGCAAACCAACCTG; CEP2 qRT reverse: TTCCACAAGATCCCGTAAACA. qRT-PCR was carried out as described [26]. Two separate mother plants were transformed and gave rise to *cep1/cep2* double ko/kd mutants 2.x or 3.x. Eleven individual homozygous lines of *cep1/cep2* double ko/kd mutants (*cep1*+*cep2-*RNAi) were screened by qRT-PCR for *CEP2* expression (S2 Fig). Line 2.21 with *CEP2*-regulation at 0.5% and line 3.14 with *CEP2*-regulation at 2% of WT were chosen for further analysis. Expression of the reporter protein pro-3xHA-mCherry-KDEL in young seedlings was below detection level using Confocal Laser Scanning Microscopy (CLSM).

*CEP1* expression in primary root cells was monitored by CLSM, using 10 days old *cep1* ko mutant seedlings containing the CEP1 reporter with the CEP1 targeting sequences fused to a three-fold hemagglutinin-tag, the green fluorescent protein EGFP and the mature CEP1 protein under control of the endogenous *CEP1* promoter (P_CEP1_::pre-pro-3xHA-EGFP-AtCEP1-KDEL, [26]). *CEP2* expression was monitored by CLSM using 10 days old WT seedlings containing the CEP2 reporter with the CEP2 targeting sequences fused to a three-fold hemagglutinin-tag, the mCherry fluorescent protein and the mature CEP2 protein under control of the endogenous *CEP2* promoter (P_CEP2_::pre-pro-3xHA-mCherry-AtCEP2-KDEL, [6]).

### Propidium iodide and calcofluor-white staining of plant tissues

Roots of 7–10 days old seedlings were mounted on a microscope slide, incubated with propidium iodide (20 μg/ml H_2_O; Sigma-Aldrich Chemie, Germany) or calcofluor-white (200 μl 500 mM Na-phosphate, 400 μl H_2_O, 400 μl calcofluor-white stain pH 7.5; Sigma-Aldrich Chemie, Germany) and examined by CLSM.

### Whole mount immunolocalization

The main procedure was done as described [30]. Seedlings were grown for 10 days on Gamborg medium. Their roots were fixed in formaldehyde (4% in 1×PBS [140 mM NaCl, 2.7 mM KCl, 4.3 mM Na_2_HPO_4_, 1.5 mM KH_2_PO_4_, pH 7.4] and 0.1% Tween) for 1h, followed by several washes with distilled water and 1×PBS. Washed roots were placed on a slide to dry at RT. The samples were subsequently rehydrated in 1×PBS followed by incubation with Driselase (2% in 1×PBS) in a humid chamber at 37°C for 45 min. After repeated washing with 1×PBS, samples were incubated in 4% IGEPAL (octylphenoxypolyethoxyethanol) and 10% DMSO in 1×PBS in a humid chamber at 37°C for 1 h, followed by several washes in distilled water and 1×PBS, then incubated for 30 min at RT with a blocking solution (2% BSA in 1×PBS and 0.01% Tween), and finally with rabbit antibody to the AtCEP2 II peptide, that is the 15 amino acids of AtCEP2 directly adjacent to the C-terminal "KDEL" ER retention signal (C-IKLSSSNPTPKDGDV, [4]) (1:60; Eurogentec, Belgium) overnight at 4°C. The preparation was washed with blocking solution, and subsequently incubated with cyanine CY2-coupled secondary anti-rabbit antibody (1:60; Dianova) at 37° C for 4 h, and finally washed with blocking solution and covered with water for microscopy with a Leica SP8 and a 40× objective using a laser intensity of 20%. The cyanine CY2-coupled secondary antibody was excited at 488 nm and emission was measured at 500–550 nm. Autofluorescence of the nuclei was recorded by excitation at 405 nm and emission at 420–475 nm.

### LR formation as synchronized by a 90° gravitropic stimulus (“root bending assay”)

The procedure was done as described [31].

### Confocal laser scanning microscopy

CLSM was carried out as described ([26], Fluoview FV 1000, Olympus, Japan) with excitation at 488 nm and emission at 503–550 nm for EGFP, excitation at 561 nm and emission at 570–630 nm for mCherry, excitation at 405 nm and emission at 420–450 nm for calcofluor and excitation at 488 nm and emission at 560–640 nm for propidium iodide. Single pictures or stacks of pictures with 0.5 μm increments at higher resolution and 2.5 μm increments at lower resolution were made. A Leica confocal laser scanning microscope (Leica SP8, Germany) was used for mCherry-CEP2 in epidermal cells to be separated for LR emergence.

## Results

### Loss of CEP2 function impairs elongation of primary root cells such as trichoblasts resulting in shortened primary roots

**Fig 1:**
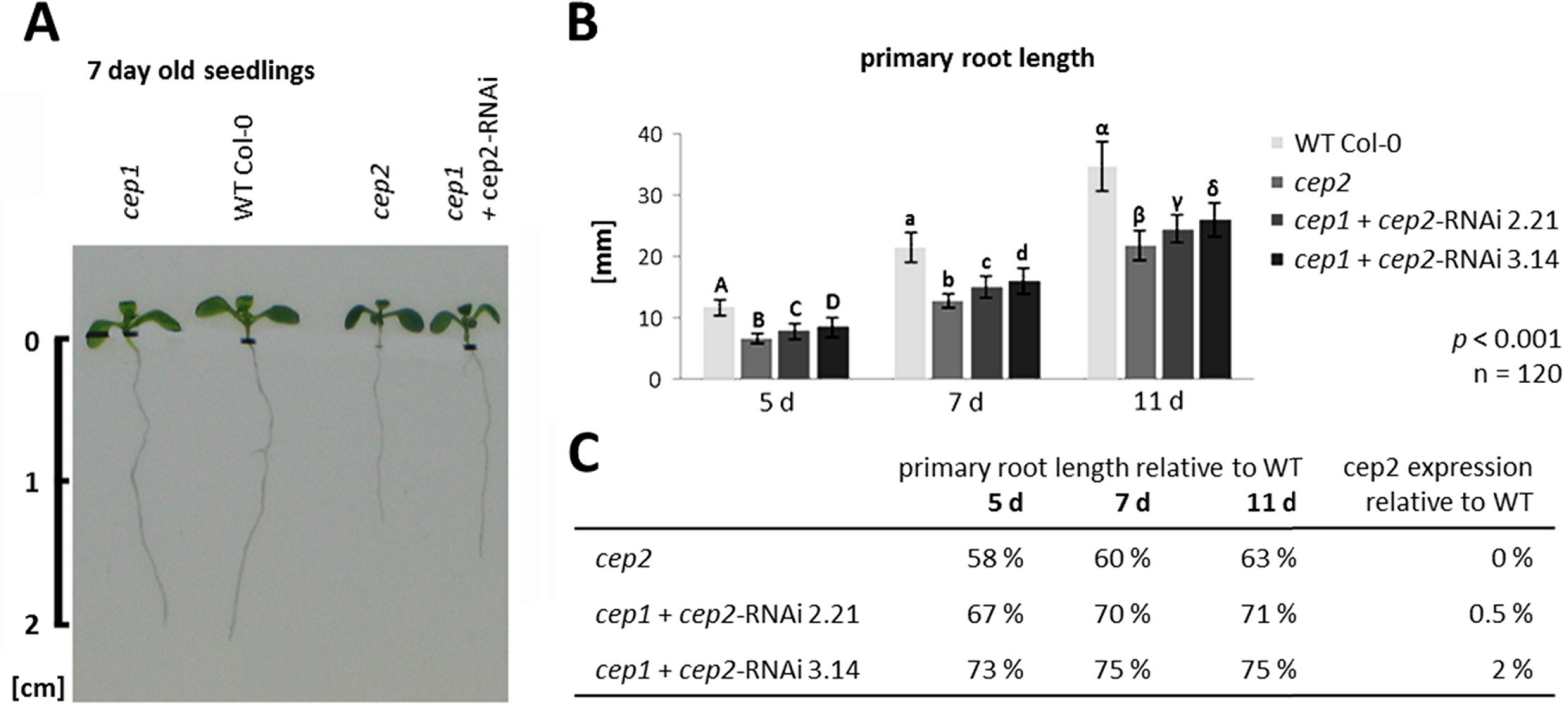
Loss of *CEP2* function results in shortened primary roots. Residual *CEP2* activity in *CEP2* knock down plants results in longer roots as compared to *cep2* knock out plants, but shorter as compared to WT. (A) Primary root length in seven days old seedlings of homozygous *cep1* ko, *cep2* ko and of *cep1 cep2* double ko/kd mutants (*cep1*+*cep2-*RNAi) versus Col-0 WT plants. *cep2* ko mutants exhibited shorter primary roots compared to Col-0 WT plants. Loss of *CEP1* has no influence on primary root length. Residual *CEP2* activity (0.5–2% compared to WT) in *cep1 cep2* double ko/kd plants resulted in primary roots significantly longer than *cep2* ko mutants but shorter than WT plants. Root length is shown in cm. (B) Comparison of primary root length in 5, 7 and 11 days old seedlings of homozygous *cep2* ko plants and *cep1 cep2* double ko/kd mutants (*cep1*+*cep2-*RNAi) with a residual *CEP2* activity of 0.5% (line 2.21) and 2% (line 3.14) as compared to WT (100% activity). Columns marked with different letters indicate statistically different groups according to the ANOVA-and Duncan test (*p*<0.001). (C) Primary root length expressed as percentage of WT plants. Data represent the respective means of two independent experiments (biological replica) each comprising 120 plants per line.

**Fig 2:**
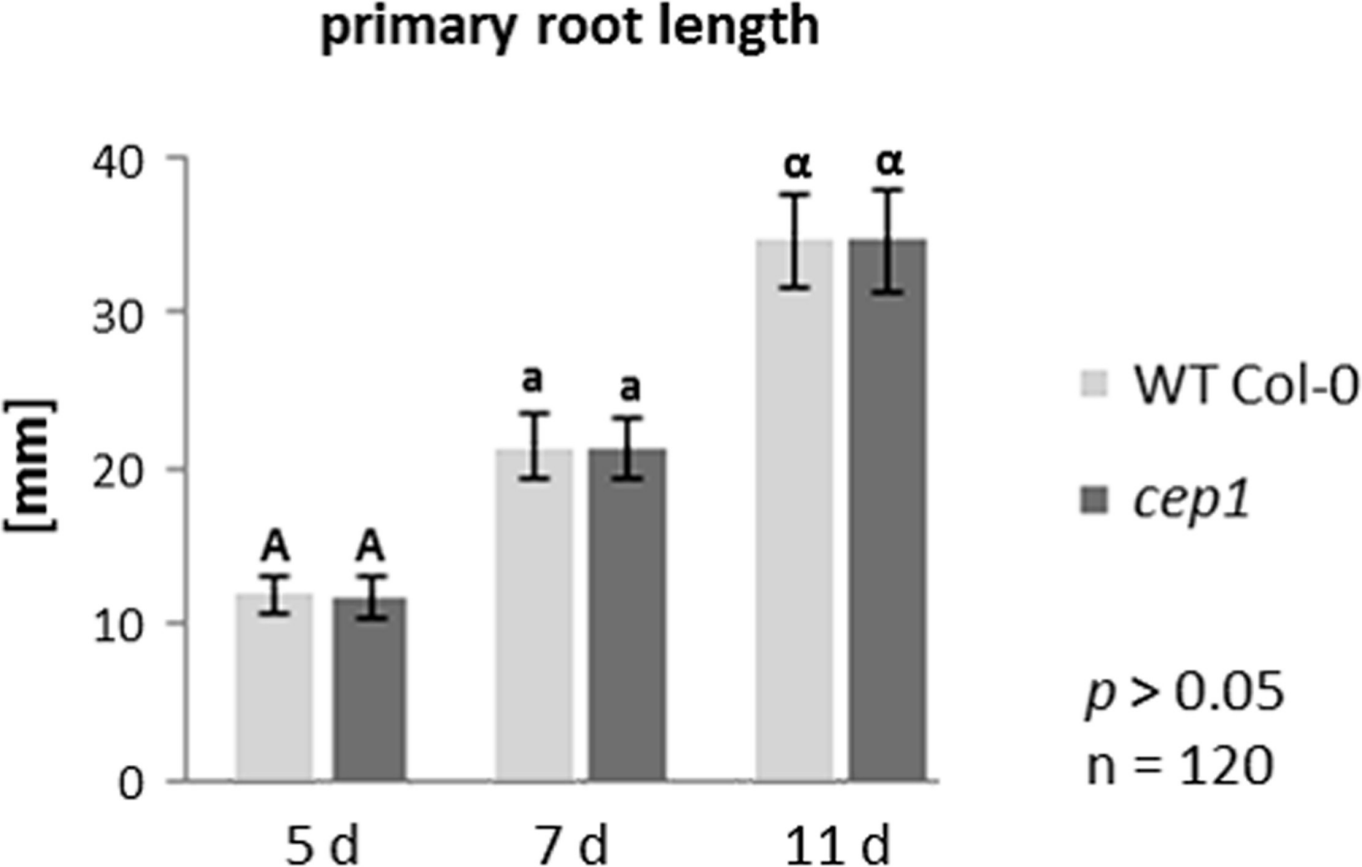
Single loss of function in *cep1* mutant plants does not result in shortened primary root length. Comparison of primary root length in 5, 7 and 11 days old seedlings of homozygous *cep1* ko mutant plants as compared to WT plants. Columns marked with similar letters indicate groups not statistically different according to the ANOVA-and Duncan test (*p*>0.05). (B) Primary root length expressed as percentage of WT plants. Data represent the respective means of two independent experiments (biological replica) each comprising 120 plants per line.

*cep*2 single ko mutants exhibited shorter primary roots compared to Col-0 WT plants, whereas the length of primary roots of the *cep1* single ko mutants was similar to Col-0 WT plants (Fig 1A). The *cep1*+*cep2-*RNAi mutant plants, which had *CEP2* expression levels that were 0.5% (line 2.21) and 2% (line 3.14) of WT (S2 Fig), also had shorter primary roots compared to Col-0 WT plants, but longer than *cep*2 ko mutants (Fig 1A). Interestingly, the residual *CEP2* activity resulted in primary roots significantly longer than the *cep*2 ko mutants and shorter than the Col-0 WT plants (*p*<0.001, Fig 1B). The primary root length of 5-, 7- and 11-days old *Arabidopsis* seedlings in *cep2* ko mutants was 58–63% of Col-0 WT, whereas the *cep1*+*cep2-*RNAi mutant with 0.5% CEP2 activity was 67–71% of Col-0 WT primary root length and the *cep1*+*cep2-*RNAi mutant with 2% CEP2 activity was 73–75% of Col-0 WT (Fig 1C). The primary root length in *cep1* single ko mutant plants exhibited no significant difference compared to WT (*p*>0.05, Fig 2). Besides the root length depending on the CEP2 activity, we observed the rosette size to be smaller comparing the entire plant of *cep2* mutants with WT. We concentrated our analysis, however, on the roots as a good model for cell elongation and directed growth.

We determined which tissue or cell type might be responsible for the shortened primary root length in *CEP2* loss of function mutants. The cells in the upper part of the lateral root (LR) cap (PCD Site I), which are the most distant to the LR cap cells near the columella root cap (PCD site II), elongate before they degrade attached to the root surface; disturbed LR cap cell elongation might influence the primary root length. Furthermore, the primary root length might be influenced through the cell length in the meristematic zone and in the elongation zone (for a general orientation see S3 Fig).

**Fig 3:**
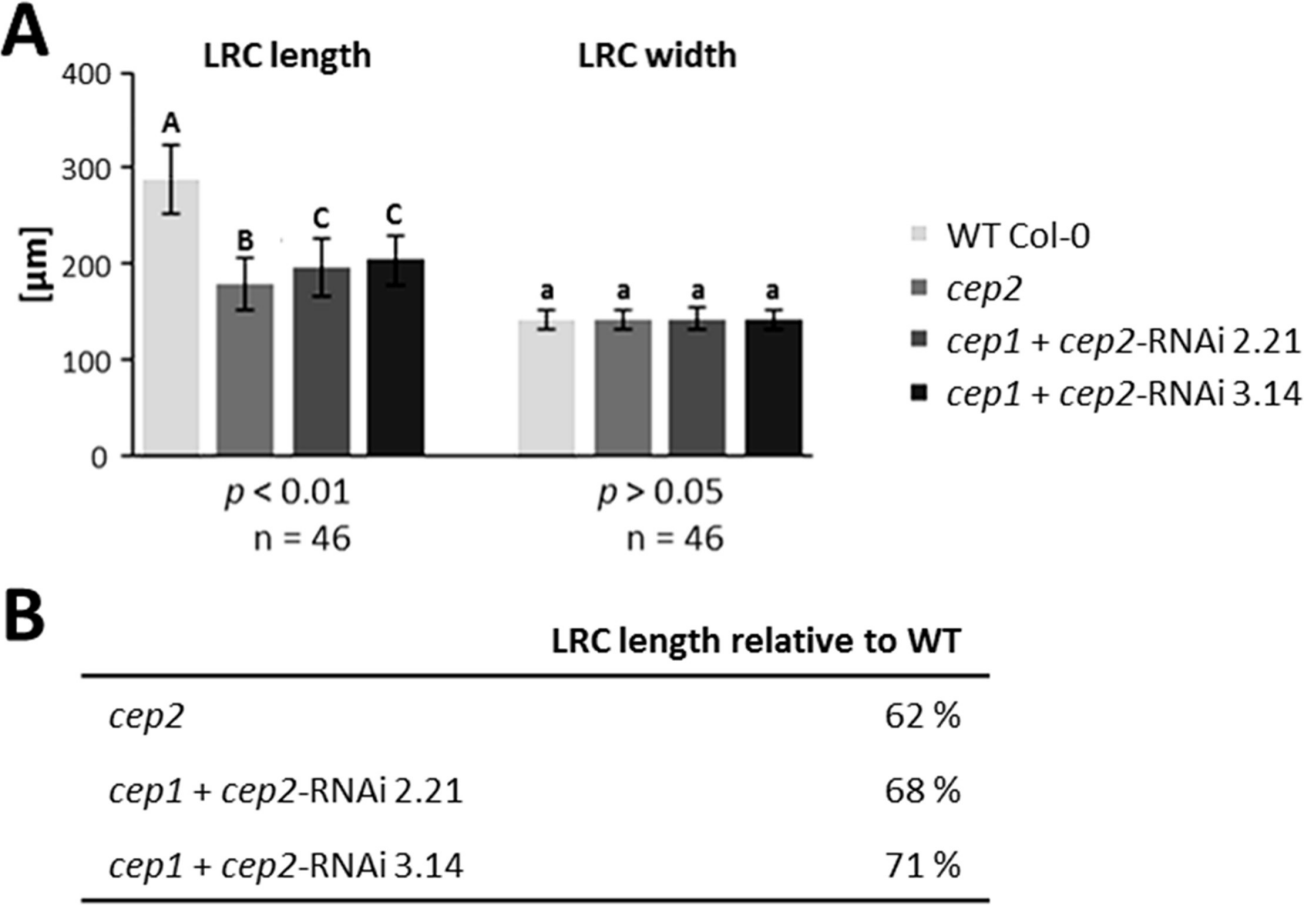
*CEP2* loss of function results in shortened lateral root cap (LRC). **The residual *CEP2* activity in *cep1 cep2* double ko/kd mutants (*cep1*+*cep2-*RNAi) plants results in a prolonged LRC as compared to *cep2* ko mutant plants, but shorter as compared to WT plants. The LRC width is not impaired.** (A) Comparison of LRC length and width in 7 days old seedlings of WT with *cep2* ko and *cep1 cep2* double ko/kd mutants (*cep1*+*cep2-*RNAi; lines 2.21 and 3.14). Columns marked with different letters indicate statistically different groups for LRC length according to the ANOVA-and Duncan test (*p*<0.01). Columns marked with similar letters indicate groups not statistically different for LRC width according to the ANOVA-and Duncan test (*p*>0.05). (B) LRC length expressed as percentage of WT plants. Data represent the respective means of 46 plants in three independent experiments (biological replica) each comprising 15 plants per line (experiment 1 and 2, each) or 16 plants (experiment 3) per genotyp.

Primary roots of 7 days old seedlings were stained with propidium iodide in order to visualize the cell wall and to measure cell dimensions in the LR cap and the elongation zone. Cells approaching PCD could also be identified since their cell membranes become permeable, resulting in propidium iodide stained nuclei in addition to the cell wall. We measured the mean length of the LR cap, which is the distance between the quiescent centre and the end of the three longest living root cap cells at PCD Site I, and the width of the LR cap at the position of these longest living root cap cells at PCD Site I (Fig 3; for a general orientation see S3 Fig). LR cap length was 289.7 μm in WT plants *versus* 179.8 μm in the homozygous *cep2* knock out mutant plants (62% of WT); 196.9 μm LR cap length (68% of WT) in the *cep1*+*cep2-*RNAi mutant line 2.21with 0.5% CEP2 activity and 204.3 μm (71% of WT) in the *cep1+cep2-RNAi* mutant line 3.14 with 2% CEP2 activity (*p*<0.01; Fig 3A left and 3B). There was no statistically significant difference in the width of the LR cap between WT plants, homozygous *cep2* ko mutants and the *cep1*+*cep2-*RNAi mutant lines (*p*>0.05; Fig 3A right).

**Fig 4:**
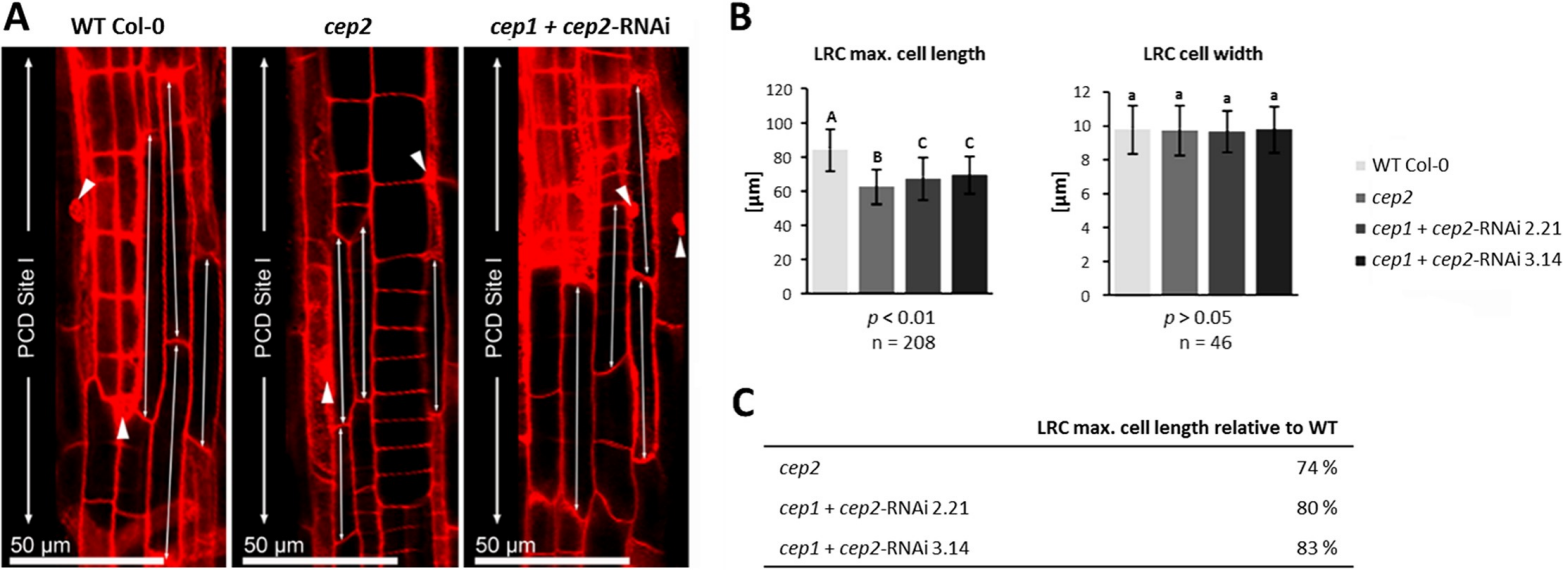
*CEP2* loss of function results in shortening the longest cells of the lateral root cap (LRC) at the level of the PCD site I. **The residual *CEP2* activity in *cep1 cep2* double ko/kd mutants (*cep1*+*cep2-*RNAi) results in prolonged LRC cells as compared to *cep2* ko mutants, but shorter as compared to LRC cells in WT plants.** (A) 7 days old seedlings of WT, *cep2* ko and *cep1 cep2* double ko/kd mutants (*cep1*+*cep2-*RNAi) were stained with propidium iodide and analyzed by CLSM (single pictures, 400-fold magnification). The 3–5 longer most cells of the LRC at the level of the PCD site I (double arrow) are compared. Nuclei stained with propidium iodide are marked (arrow heads) indicating cells approaching PCD since their cell membranes become permeable resulting in propidium iodide stained nuclei in addition to the stained cell wall. (B) Comparison of the length (left) and the width (right) of the 3–5 longer most LRC cells in 7 days old seedlings of WT plants with *cep2* ko and *cep1 cep2* double ko/kd mutants (*cep1*+*cep2-*RNAi; lines 2.21 and 3.14) mutants. Columns are marked with different letters indicating statistically different groups for LRC cell length (*p*<0.001) or with similar letters indicating groups not statistically different for LRC cell width (*p*>0.05) according to the ANOVA-and Duncan test. Data represent the respective means of 208 plants in three independent experiments (biological replica) each comprising 15 seedlings per line (experiment 1 and 2, each) or 16 plants (experiment 3) per genotype resulting in 208 longer most LRC cells (3–5 cells per LRC) evaluated for the cell length and 46 LRC cells evaluated for the cell width. (C) LRC cell length expressed as percentage of WT plants.

In general, the longest cells in the LR cap are found at PCD Site I (Fig 4; S3 Fig, asterisk). The longest LR cap cells (the 3–5 LR cap cells exhibiting max. cell length) of *cep2* ko mutants (50–60 μm) were shorter than the longest LR cap WT cells (75–90 μm); the longest LR cap cells of the *cep1*+*cep2-*RNAi (60–75 μm) were also shorter than WT cells, but longer than in *cep2* ko mutants (50–60 μm) (Fig 4A, double arrows). PCD of LR cap cells in *cep2* ko mutants was similar to WT as indicated by the nuclei of these cells stained–in addition to the cell wall—with propidium iodide indicating perforated cell membranes and thus the beginning PCD (Fig 4A, arrowheads). This observation was confirmed by statistical evaluation of the 3–5 longest cells in the LR cap (*p*<0.001; Fig 4B and 4C). The mean length of Col-0 WT LR cap cells was 83.9 μm, *versus* 62.4 μm (74% of WT) in *cep2* ko mutants, and *versus* 67.1 μm (80% of WT) in the *cep1*+*cep2-*RNAi mutants with 0.5% *CEP2* activity and 69.3 μm (83% of WT) with 2% *CEP2* activity (Fig 4B left and 4C). There was no statistically significant difference in the mean width of the longest LR cap cells between WT and *cep2* mutant plants (*p*>0.05; Fig 4B right).

**Fig 5:**
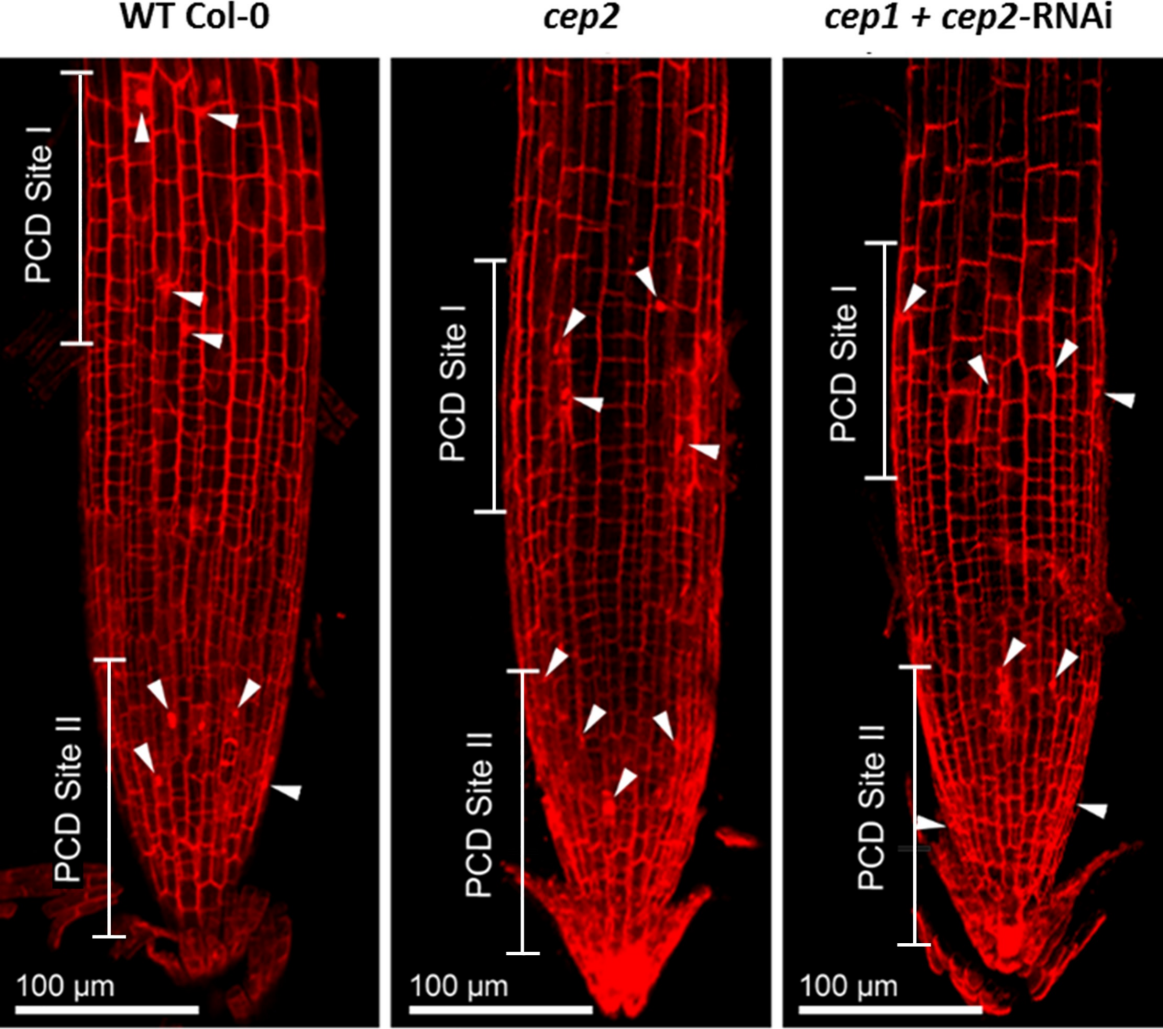
*CEP2* loss of function does not impair PCD in the LRC, although the PCD site I is shifted more close to the root tip in *cep2* ko mutants due to the reduced cell lengh of the LRC and again to a lesser extent in *cep1 cep2* double ko/kd mutants as compared to WT plants. 7 days old seedlings of WT, *cep2* ko and *cep1 cep2* double ko/kd mutants (*cep1*+*cep2-*RNAi) were stained with propidium iodide and analyzed by CLSM (stack of 14 optical sections, 200-fold magnification). PCD site I and II are indicated by nuclei stained with propidium iodide (arrow heads) thus indicating cells approaching PCD since their cell membranes become permeable to propidium iodide.

PCD in the LR cap was similar in WT plants, in *cep2* ko mutants and in *cep1+cep2-*RNAi mutants, giving rise to normal development of the LR cap (Fig 5). The lengths of the PCD Sites I and II in *cep2* ko mutants and *cep1*+*cep2-*RNAi mutants were similar to WT plants (Fig 5) and both PCD sites can be recognized as a distinct zone containing cells with propidium iodide stained nuclei and cell walls (Fig 5, arrowheads). However, the PCD Site I was closer to the root tip due to the reduced cell length of the LR cap in *cep2* ko mutants and again to a lesser extent in *cep1+cep2*-RNAi mutants compared to WT plants (Fig 5). The general morphological organization of the *Arabidopsis* root tip is depicted in S3 Fig. The different PCD sites represent the edges of the respective root cap layers that change over time. At about 5 days after germination, there are two PCD sites, PCD site II is formed by the oldest root cap layer in which PCD has already moved towards the columella, whereas PCD site I is formed by the next younger root cap layer which still covers the entire meristem, so that the PCD happens close to the elongation zone. As these sites are not fixed but move in dependency of the age of the root imaged [8], slight differences between the images in Fig 5 and the model in S3 Fig are unavoidable.

**Fig 6:**
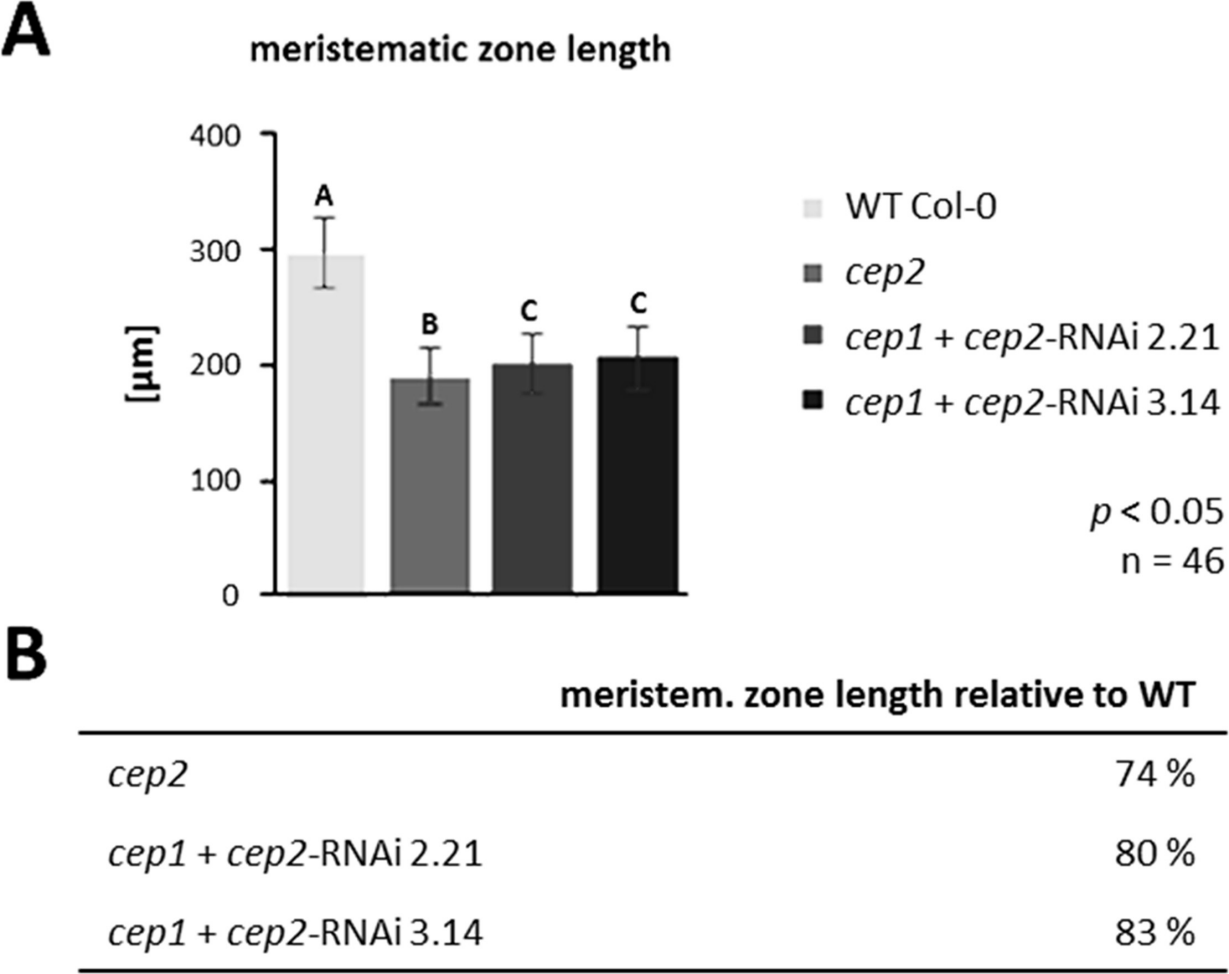
*CEP2* loss of function results in shortening the length of the meristematic zone. **The residual CEP2 activity in *cep1 cep2* double ko/kd mutants (*cep1*+*cep2-*RNAi) results in a slightly prolonged meristematic zone as compared to *cep2* ko mutant plants, but shorter as compared to WT plants.** (A) Comparison of the meristematic zone length in 7 days old seedlings of WT with *cep2* ko and *cep1 cep2* double ko/kd mutants (*cep1*+*cep2-*RNAi; lines 2.21 and 3.14) mutants. Columns marked with different letters indicate statistically different groups according to the ANOVA-and Duncan test (*p*<0.05). (B) Meristematic zone length expressed as percentage of WT plants. Data represent the respective means of 46 plants in three independent experiments (biological replica) each comprising 15 plants per line (experiment 1 and 2, each) or 16 plants (experiment 3) per genotyp.

The mean length of the meristematic zone, which is the distance between the quiescent centre and the first elongated epidermis cells (Fig 6; for a general orientation see S3 Fig), was 296.2 μm in Col-0 WT plants; 188.7 μm (64% of the WT) in *cep2* ko mutants; 201.2 μm (68% of the WT) in *cep1*+*cep2*-RNAi line 2.21 mutants and 206.3 μm (70% of the WT) in *cep1*+*cep2*-RNAi line 3.14 mutants (*p*<0.05; Fig 6). Meristem length and root cap length are correlated in both mutant and WT plants.

**Fig 7:**
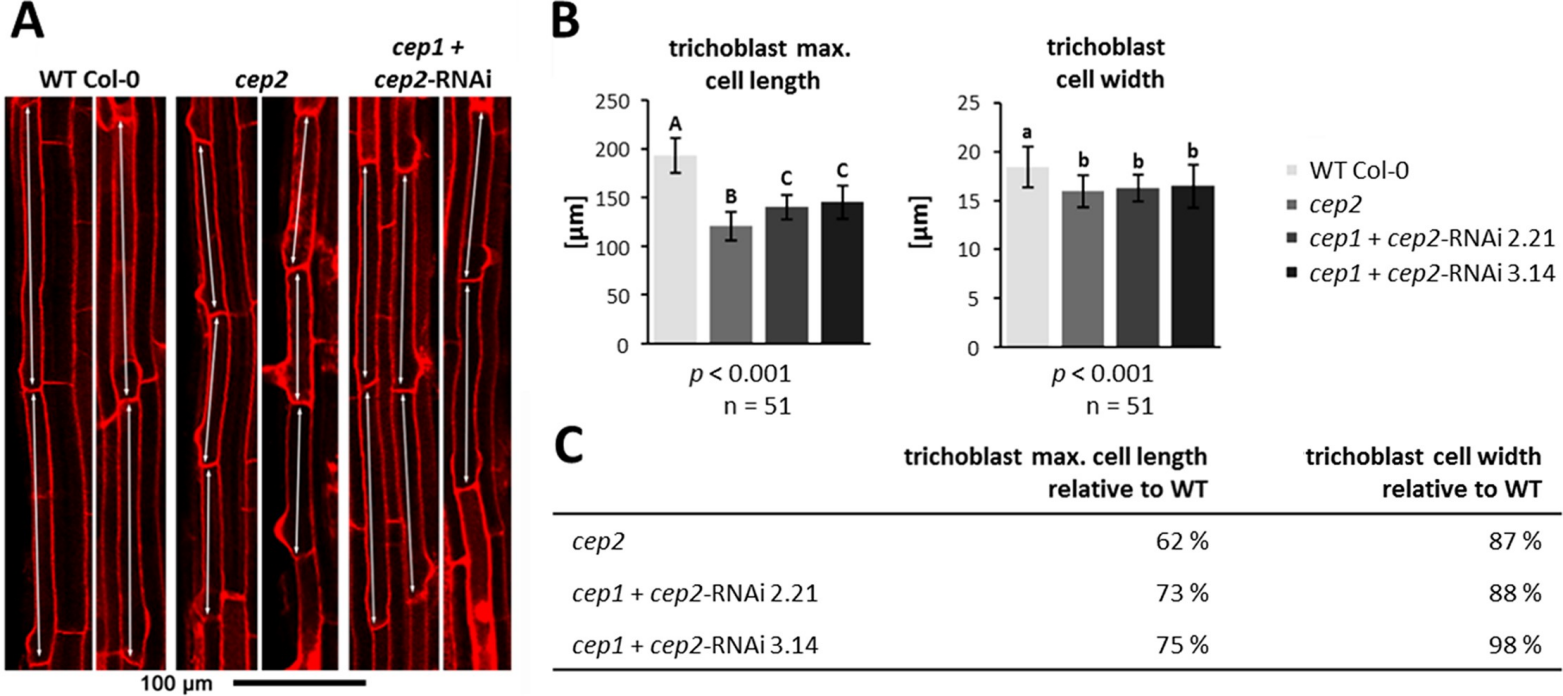
*CEP2* loss of function results in shortening the cell length and cell width of the longest trichoblasts (double arrows) in the root elongation zone at the level of the transition from the rapid elongation zone to the late elongation zone. **The residual *CEP2* activity in *cep1 cep2* double ko/kd mutants (*cep1*+*cep2-*RNAi) results in prolonged trichoblasts as compared to *cep2* ko mutant plants, but shorter as compared to trichoblasts in WT plants.** (A) 7 days old seedlings of WT, *cep2* ko and *cep1 cep2* double ko/kd plants were stained with propidium iodide and analyzed by CLSM (single pictures, 200-fold magnification). Two examples of propidium iodide stained roots are presented for each genotype. (B) Comparison of the trichoblast cell length (left) and the width (right) in 7 days old seedlings of WT plants with *cep2* ko and *cep1 cep2* double ko/kd mutants (*cep1*+*cep2-*RNAi; lines 2.21 and 3.14). Columns are marked with different letters indicating statistically different groups for the trichobalst cell length or with similar letters indicating groups not statistically different for trichoblast cell width according to the ANOVA-and Duncan test (*p*<0.001). (C) Trichoblast cell length expressed as percentage of WT plants. Data represent the respective means of two independent experiments (biological replica) comprising 8 and 9 seedlings per line, respectively, with three trichoblast cells evaluated per seedling.

We concentrated our analysis of CEP2 involvement in cell elongation on young trichoblasts (the elongated cells developing root hairs) as an example for epidermis cells, since they are localized at the end of the rapid elongation zone and can easily and unequivocally be identified through their emerging protrusions of root hairs. We calculated the mean cell length and width of the three longest trichoblasts in the elongation zone at the level of the transition from the rapid elongation zone with rapid cell elongation to the late elongation zone, where cell elongation is no longer increasing. The transition between the rapid and late elongation zones can be recognized by the formation of the emerging protrusions of root hairs in trichoblasts (Fig 7A; for a general orientation see S3 Fig). The trichoblasts in WT plants (180–200 μm) were statistically significantly longer than in *cep2* ko mutants (110–130 μm) and in *cep1+cep2*-RNAi mutants (135–155 μm) (*p*<0.001; Fig 7A, double arrows, 7B left and 7C). The cell width of the three longest trichoblasts of the *cep2* ko mutants also was significantly smaller than in WT plants (*p*<0.001; Fig 7B right and 7C) with a significantly smaller total root width at the elongation zone as compared to WT that parallels the trichoblast cell width (*p*<0.01; Parts A and B in S4 Fig). Loss of *CEP2* alone results in trichoblasts with a width of 87% as compared to WT and thus with 87% an equally reduced width of the elongation zone. Residual *CEP2* activity in *cep1/2* double ko/kd lines resulted in 88–89% smaller trichoblasts and elongation zone as compared to WT. There is no indication that CEP2 activity is restricted to trichoblasts. The elongation of other cells in the epidermis such as atrichoblasts, or cortex cells might also be impaired.

CEP2 controls the growth of primary roots by influencing epidermal cell elongation. We therefore tried to localize the expression of CEP2.

### Pro-CEP2 resides in the ER of root cap cells prepared for PCD and is still present in root cap corpses

**Fig 8:**
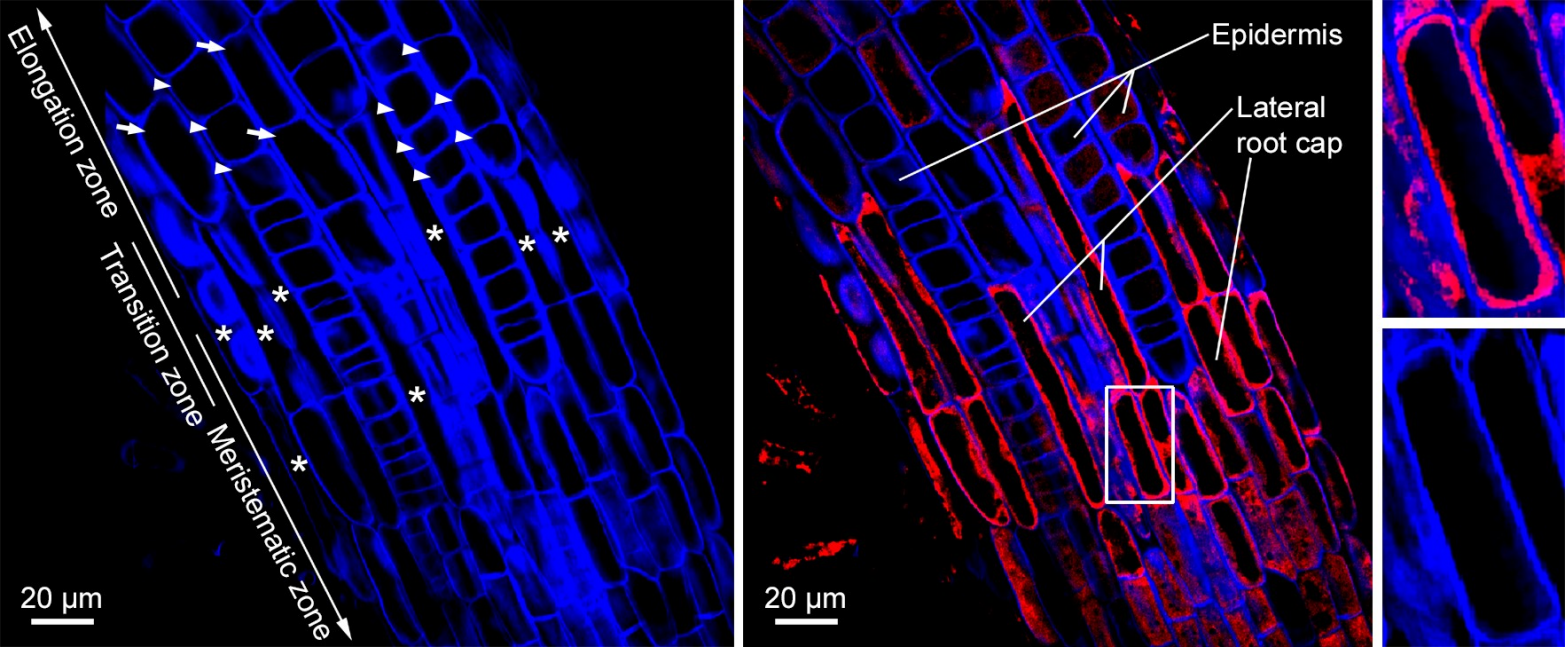
Pro-CEP2 accumulates within the protoplasm of the longer most cells from the upper end of the LR cap (PCD site I) at the level of the transition zone and is still present in root cap corpses. Roots of 7 days old WT seedlings expressing the pro-mCherry-CEP2 (P_CEP2_::pre-pro-3xHA-mCherry-AtCEP2-KDEL) are stained with calcofluor-white in order to visualize the cell wall and analyzed by CLSM (stack of 4 optical sections, Olympus Fluoview FV 1000). Left: Visualization of cell walls (blue): isodiametric, not elongated epidermis cells (arrow heads) from the meristematic and transition zone as well as already elongating epidermis cells (arrows) from the rapid elongation zone in addition to small, elongated and already collapsing cells at the upper end of the LRC (asterisk) are found. Middle: Merge of calcofluor (blue) and pro-mCherry-CEP2 (red): pro-CEP2 is found in the protoplasm of LR cap cells and is still present in root cap corpses sticking to epidermis cells of the rapid elongation zone. Pro-CEP is localized within the cell in the protoplasm surrounding the large vacuole and not in the cell wall. Right: magnification of the white boxed area presenting a LR cap cell with the blue coloured cell wall, the large black looking vacuole and in between the protoplasm with the red coloured pro-CEP2.

Calcofluor-white was used to visualize the cell walls of the roots of young seedlings transformed with the reporter construct *P*_*CEP2*_::*pre-pro-3xHA-mCherry-AtCEP2-KDEL* in the Col-0 WT background (Fig 8). Within the transition zone, there were isodiametric, non-elongated epidermal cells from the meristematic zone (Fig 8 left, arrowheads) and elongating epidermal cells (Fig 8 left, arrows) indicating the beginning of the elongation zone. There were also small, elongated cells (Fig 8 left, asterisk) representing collapsing cells from the upper end of the LR cap at PCD site I in the process of degrading while still attached to epidermal cells. Typically, the width of root cap cells is half the width of the underlying epidermis cells. Pro-3xHA-mCherry-AtCEP2-KDEL was seen in LR cap cells prepared for PCD at the upper end of the LR cap and accumulated in the cytoplasm surrounding the vacuole (Fig 8, middle and right); typically, pro-CEP accumulate within the ER and ER derived organelles [6, 26]. After death of LR cap cells, mCherry signal appears to localize to the degrading remains of LR cap cells (Fig 8, middle): dotted red signals on the left side of the root within the transition zone and on the right side of the root left to the word “Epidermis” and “Lateral”. The faint dotted and weak red signals within epidermal cells (Fig 8, middle: upper part) could be interpreted as unspecific back ground signals of pro-3xHA-mCherry-AtCEP2-KDEL. However, even if we do find CEP2 expression in the epidermal cells of the meristematic zone, the transition zone, or the elongation zone, it would be much lower than in the root cap.

### CEP2 was immuno-localized to root cap corpses sticking to epidermis cells in the rapid elongation zone

**Fig 9:**
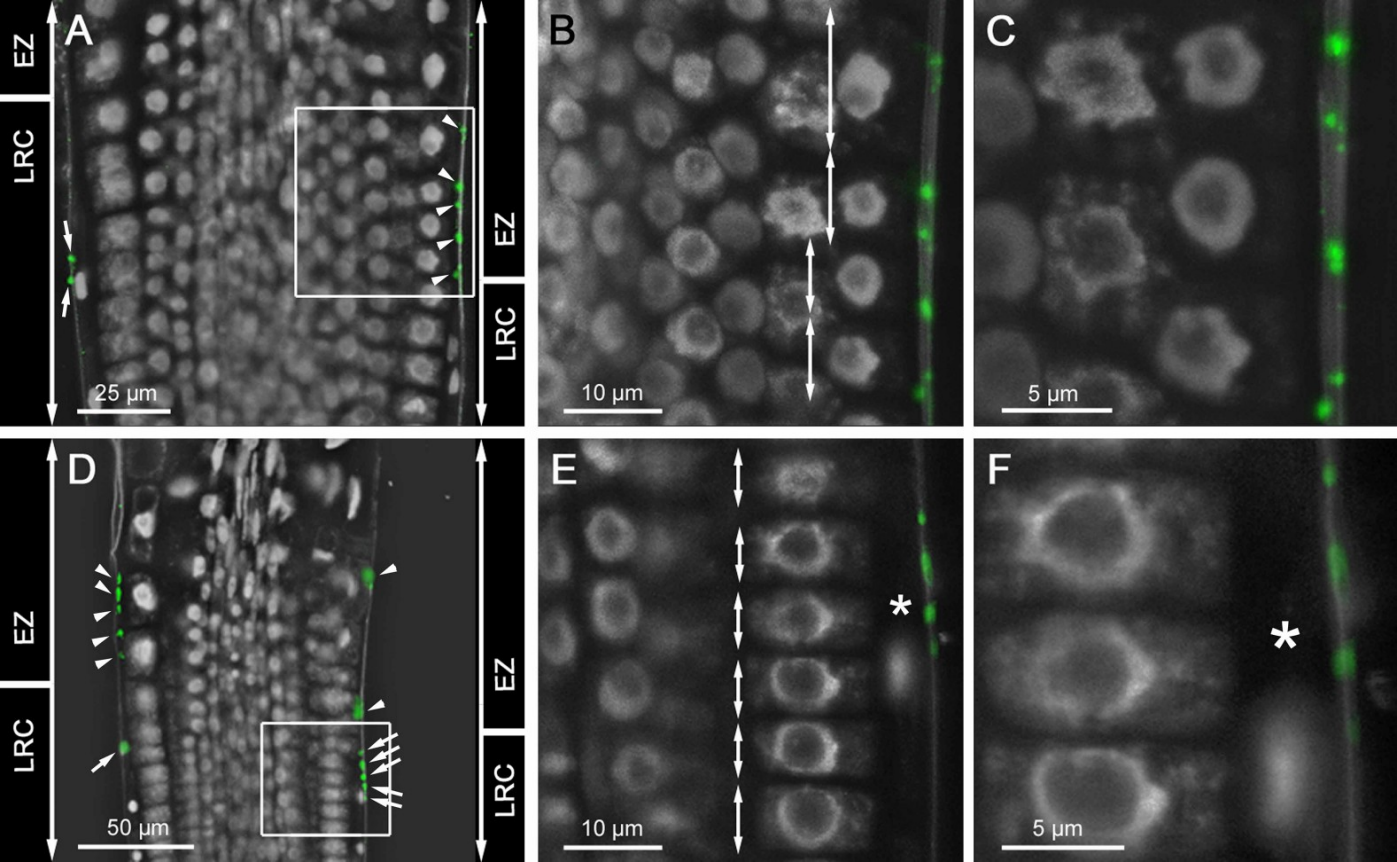
CEP2 is immunolocalized to root cap corpses sticking to epidermis cells in the rapid elongation zone. (A and D) Two root tips of 10 days old WT seedlings incubated with anti-CEP2 peptide antibody and cyanine CY2-coupled secondary antibody are analyzed by CLSM (Leica SP8). Nuclei are characterized by their strong white auto fluorescence, whereas other parts of the cell are barely fluorescing and look black. (A-C) is focusing on epidermis cells of the elongation zone (EZ), whereas (D-F) is focusing on cells at the upper end of the LR cap (LRC) with two different magnifications of the white boxed area in (B-C) and (E-F), respectively. LR cap cells are long and small with one single nucleus (see asterix in E and F), whereas epidermis cells are short at the level of the LR cap and are elongating at the level of the rapid elongation zone (compare double arrows in B and E). CEP2 signals (green) are exclusively found in LR cap cells (see arrows in A and D) and in root cap corpses sticking to the cell wall of epidermis cells at the beginning elongation zone (see arrow heads in A and D).

Maturation of pro-CEP2 to the enzymatically active protein by acidification results in autocatalytic cleavage of the pro-peptide and the KDEL-motif plus cleavage between mCherry and the mature CEP2 protein [6]. As a consequence, the mature CEP2 protein cannot be detected by CLSM. For that reason, whole mount immunolocalization with anti-peptide antibodies recognizing the 15 amino acids directly upstream of the C-terminal KDEL-motif (C-IKLSSSNPTPKDGDV; [6]) was used to localize mature CEP2 to cells at the upper end of the LR cap prepared for PCD (PCD site I) just below the transition zone and possibly to epidermal cells at the beginning elongation zone and the rapid elongation zone in the root tip of young seedlings (Fig 9). LR cap cells are very elongated with one small nucleus and are much longer than the cells beneath in the meristematic zone (Fig 9A and 9D; see 9E and 9F, asterisk). The distinct punctate CEP2 signals were localized exclusively in corpses of LR cap cells (Fig 9A and 9D arrows; 9E and 9F). The epidermal cells beneath were short (Fig 9E, double arrows) with their nucleus in the centre of the cell; they exhibited no CEP2 signal. As soon as the LR cap cells collapsed due to PCD, the length of the epidermal cells increased (Fig 9B, double arrows) and their nuclei are localized to the inner surface of the epidermal cell (Fig 9B and 9C). Both observations identify the transition between the end of the LR cap and the beginning of the elongation zone, the rapid elongation zone [32]. Within the transition zone, the distinct punctate CEP2 signals are localized exclusively to root cap corpses still attached to epidermal cell walls (Fig 9A and 9D arrow heads, 9B and 9C). This observation corresponds to the pro-3xHA-mCherry-CEP2-KDEL localization in the degrading remains of the dead LR cap cells (Fig 8, middle).

As negative controls, no background signal was detected in the *cep2* ko mutant using the same antibody or in Col-0 WT plants using pre-immune serum (not shown). We detected specific CEP2 signals in whole mount immunolocalization within the differentiating protoxylem of the root tip vasculature (S5 Fig) indicating that the antibody could penetrate all cell layers. The antibody binds only within the stele and not in epidermis cells. In a differentiated root, we found CEP2 signals in epidermis cells that have to be moved for the emerging lateral root (see next paragraph).

The loss of function mutant phenotype demonstrates that CEP2 activity is required for epidermal cell elongation as we could demonstrate it for the trichoblasts. The CEP2 activity as it is localized in root cap corpses might impact epidermal cell elongation.

### *CEP1* and *CEP2* are expressed in the root epidermis cells at lateral root (LR) emergence sites

**Fig 10:**
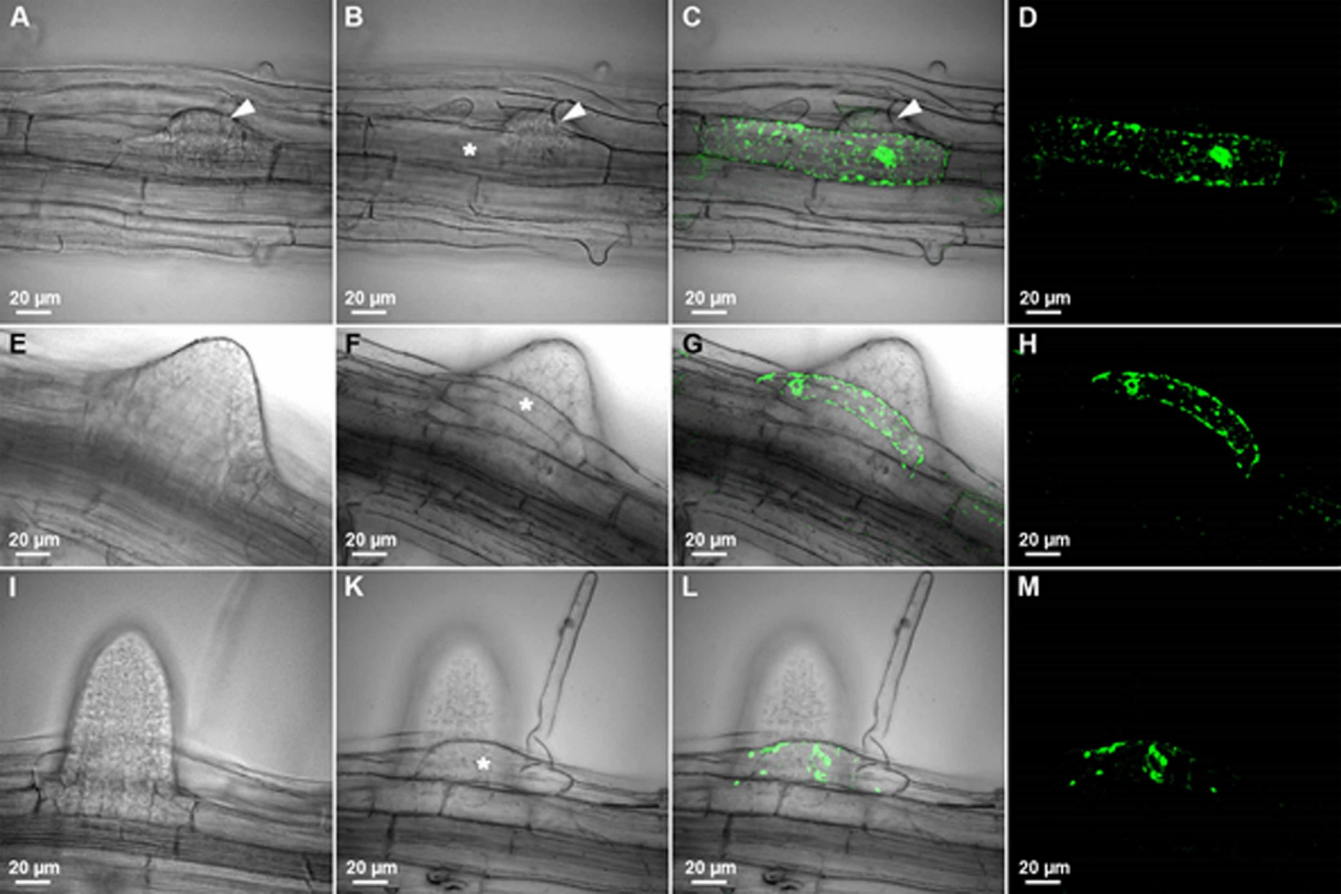
CEP1 is expressed in the root epidermis cells at lateral root (LR) emergence sites. The eight stages (roman numbers, see S6 Fig) of LR primordium development in the roots of 10 day old *cep1* ko mutant seedlings expressing pro-EGFP-CEP1 under the control of the endogenous promoter (*P*_*CEP1*_::*pre-pro-3xHA-EGFP-AtCEP1-KDEL*) were analyzed *in vivo* by CLSM (Olympus Fluoview FV 1000). No EGFP-CEP1 expression could be observed from developmental stage I-VI. (A-D) Stage VII of LR primordium development. (E-H) Stage VIII of LR primordium development. (I-M) Stage LR exhibiting the adult lateral root in LR primordium development. Arrow head in A, B and C indicates the LR primordium just emerging and separating the epidermis cells. The asterisk in B, F and K marks the epidermis cell expressing pro-EGFP-CEP1 at LR emergence sites. A, E and I: Focus is on the LR primordium. B-D, F-H, K-M: Focus is on the epidermis cells. A, B, E, F, I, K: Differential interference contrast. D, H, M: EGFP; C, G, L: merge.

LRs originate from the pericycle cells situated in front of the xylem poles. *Arabidopsis* LR emergence requires the new primordia to break through the three overlying outer tissues that are each composed of one layer of endodermis, cortex, and epidermis (see S6 Fig; [33, 34]). The involvement of CEP1 in the cell separation necessary for LR emergence was investigated in young seedlings expressing the CEP1 reporter protein (P_CEP1_::pre-pro-3xHA-EGFP-AtCEP1-KDEL; Fig 10) *in vivo* by CLSM. No pro-EGFP-CEP1 expression was observed from developmental stage I to VI (not shown). From developmental stage VII on, when the newly formed LR primordium reaches the epidermis (S6 Fig), pro-EGFP-CEP1 was detected in the epidermis cell (Fig 10A–10D, asterisk) that must separate to make room for the emerging LR primordium (Fig 10A–10D, arrowhead). The expression of pro-EGFP-CEP1 in this epidermal cell was observed at developmental stage VIII, when emergence of the LR primordium through the epidermis is complete (Fig 10E–10H) and at later developmental stages, when the LR primordium had emerged from the epidermis and is defined as an adult LR (Fig 10I–10M). This pro-EGFP-CEP1 expressing epidermis cell has a strong curvature probably due to the mechanical forces during cell separation in the course of tissue remodelling (Fig 10). Interestingly, in this case only one cell had to be moved, perhaps resulting in the observed strong curvature. In other cases, more than one cell had to be moved, depending on the position of the emerging LR. CEP1 expression was observed in the epidermal cell next to the epidermal cell with the strong curvature (Fig 10G) indicating that the epidermal cells in the direct neighbourhood are also involved in giving room for the emerging LR.

**Fig 11:**
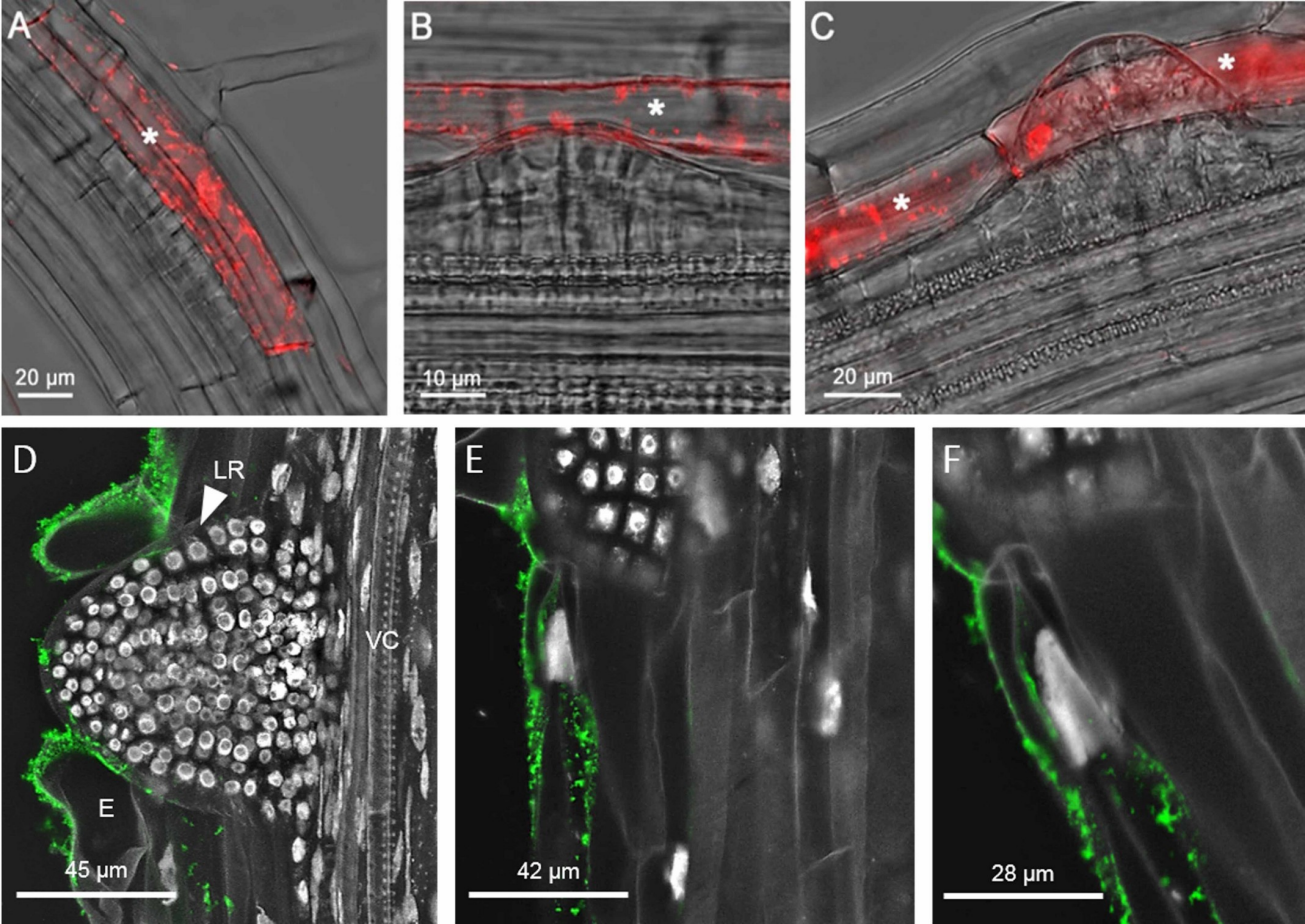
CEP2 is expressed in the root epidermis cells at lateral root (LR) emergence sites. (A-C) The eight stages (roman numbers, see S6 Fig) of LR primordium development in the roots of 10 day old WT seedlings expressing the pro-mCherry-CEP2 reporter under the control of the endogenous promoter (*P*_*CEP2*_::*pre-pro-3xHA-mCherry-AtCEP2-KDEL*) were analyzed *in vivo* by CLSM (Leica SP8). (A) Stage I-VI of LR primordium development. (B) Stage VII of LR primordium development. (C) Stage VIII of LR primordium development. The white asterisk indicates epidermis cells expressing pro-mCherry-AtCEP2 for LR emergence. (D-F) CEP2 was immunolocalized to epidermis cells that have to be moved for the emerging LR. (D) Maximum projection of LR emergence; LR, lateral root; VC, vascular cylinder; E, epidermis. (E) Single optical section shows CEP2 accumulation only in the epidermal cell bordering the LR outbreak. (F) Magnification of epidermal cell next to LR outgrow.

We also analysed young seedlings expressing the CEP2 reporter protein (*P*_*CEP2*_::*pre-pro-3xHA-mCherry-AtCEP2-KDEL*) *in vivo* by CLSM (Fig 11A–11C). Pro-mCherry-CEP2 was localized in root epidermal cells that separate to make room for the emerging LR primordium (Fig 11A–11C, asterisk). Pro-mCherry-CEP2 continued to be expressed after LR emergence, as seen for pro-EGFP-CEP1. Furthermore, CEP2 was immunolocalized to epidermis cells that have to be moved for the emerging LR (Fig 11D–11F). Similar to *CEP1* expression, *CEP2* expression was detected in the two cells that have to give room for the emerging LR and in the epidermis cells next to lateral root outgrow in order to make the cells in the direct vicinity more flexible.

The CLSM used to generate the data displayed in Fig 11 (Leica SP8) is more sensitive as compared to the CLSM used for the data presented in Fig 10 (Olympus Fluoview FV 1000), allowing for the detection of pro-mCherry-CEP2 (Fig 11A–11C), and this detection even at stages I-VI of LR primordium development.

### Loss of function of *CEP1* or *CEP2* retards lateral root (LR) emergence

**Fig 12:**
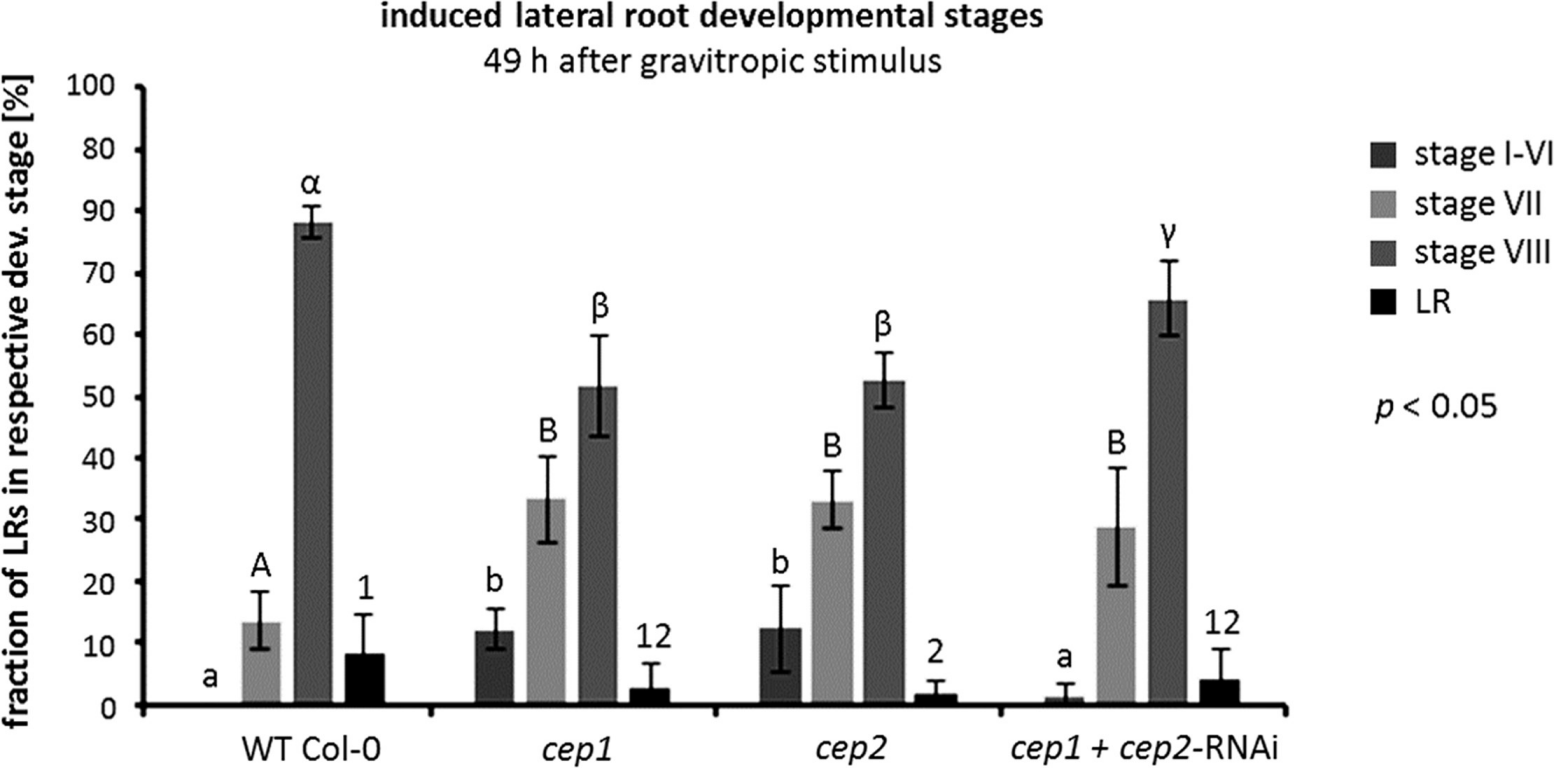
Loss of function of *CEP1* or *CEP2* retards lateral root (LR) emergence. The quantitative evaluation of the eight stages (roman numbers, see S5 Fig) of LR primordium development in the roots of 10 day old *cep1* ko, *cep2* ko, and *cep1/2* double ko/kd mutants (*cep1*+*cep2-*RNAi) were analyzed and compared to WT plants 49 hours after initiating the LR formation by “root bending”, when most of the WT plant LR primordia just reached the epidermis (stage VII). Single loss of function results in the strongest quantitative phenotype in retardation of LR development as compared to WT. Double loss of function does not result in an enhanced retardation of LR emergence. Columns represent the respective means expressed as percentage of LR primordia in the respective developmental stage in four independent experiments (biological replica): WT (15, 20, 18, or 20 plants per biological replica), *cep1* ko (26, 24, 30, or 27 plants), *cep2* ko (25, 29, 26, or 28 plants), *cep1/2* double ko/kd mutants (*cep1*+*cep2-*RNAi) (21, 24, 22, or 26 plants). Columns marked with different letters indicate statistically different groups according to the ANOVA-and Duncan test (*p*<0.05).

LR formation was synchronized by a 90° gravitropic stimulus (“bending assay”, [31]), which is a 90° rotation of 7–10 days old seedlings on an agar-plate inducing a synchronous formation of LRs at the bending site, in order to investigate if the loss of CEP function delayed LR emergence. Eight stages of LR primordium development are defined (S6 Fig). *Arabidopsis* roots originate from pericycle founder cells and undergo several rounds of anticlinal and periclinal divisions resulting in a dome-shaped primordium that penetrates the endodermis and cortex (stages I-VI) until it reaches the epidermis (stage VII). It eventually emerges from the parental root (stage VIII) becoming an adult LR. Since CEP1 and CEP2 were found in root epidermal cells, we performed the quantitative evaluation of the stages in LR primordium development 49 hours after “root bending”, when most of the WT plant primordia had just reached the epidermis (stage VII) or emerged from the parental root (stage VIII), thus combining the evaluation of stages I-VI (Fig 12).

WT plants exhibited no LR primordia in developmental stages I-VI, 14% LR primordia at developmental stage VII, 78% LR primordia emerging from the parental roots at developmental stage VIII; and 8% adult LR.

*cep1* ko or *cep2* ko mutants exhibited 12% LR primordia in developmental stages I-VI; they showed significantly more LR primordia (33%) than WT plants at developmental stage VII; *cep* single loss of function mutants had significantly fewer (*p*<0.05) LR primordia (52–53%) emerging from the parental root than WT plants at developmental stage VIII. Finally, 49 hours after root bending *cep2* single ko mutants had with 2% adult LR significantly fewer adult roots at developmental stage VIII than WT plants (*p*<0.05); *cep1* ko (3% LR) exhibited no significant difference to WT plants or to *cep2* single ko mutants.

*cep1/2* double ko/kd mutants (*cep1*+*cep2-*RNAi) mutants did not show an additive phenotype. They had 1–2% LR primordia in stages I-VI, which was more than WT plants, but less than the single *cep* ko plants (*p*<0.05). At developmental stage VII, *cep1/2* double ko/kd mutants with 29% primordia had more LR primordia than WT plants, but the difference between *cep* single ko plants and *cep1/2* double ko/kd mutants was not statistically significant (*p*<0.05). At developmental stage VII, the *cep1/2* double ko/kd mutants with 66% LR primordia emerging from the parental roots exhibited a phenotype quantitatively between single *cep* ko and WT plants. Finally, *cep1/2* double ko/kd mutants (4% adult LR) exhibited no significant difference to WT plants or to *cep2* single ko mutants.

Single loss of function of *CEP1* or *CEP2* resulted in the strongest quantitative phenotype in retardation of LR emergence. Compared to WT plants, there was a clear retardation in LR emergence resulting from *cep* single loss of function. *cep* double loss of function did not show an enhanced retardation of LR emergence compared with *cep* single loss of function. We conclude that CEP1 and CEP2 contribute to the emergence of LR primordia from the parental root by acting within the root epidermal cells.

## Discussion

The *Arabidopsis* genome encodes three KDEL-CysEPs—*AtCEP1*, *AtCEP2*, and *AtCEP3*. In this study, we investigated the role of CEP during root development using *A*. *thaliana* seedlings as a model system.

We have demonstrated that CEP2 is involved in elongation of the primary root. Loss of *CEP2* results in shortened primary roots due to reduced cell length of epidermal cells such as trichoblasts. Atrichoblasts and cortex cells might show similar elongation phenotypes. We cannot exclude that CEP2 is involved in general cell elongation resulting in an overall smaller growth of *cep2* mutant plants. Loss of *CEP2* also leads to a decrease in LR cap cell length at the level of PCD site I. The low level of *CEP2* in two independent *cep1/2* double ko/kd lines is sufficient to cause a measurable shortening of LR cap cells and the epidermal cells at the starting, rapid elongation zone of primary roots. The length of the *Arabidopsis* primary root is entirely dependent on cell extension in the elongation zone [8, 35]. Loss of *CEP2* in these genotypes results in a decreased root diameter rather than root compression, which could have influenced root length [36–38]. Thus, the shorter LR cap likely has no influence on primary root length; the root elongation is not hindered by the LR cap. The shortened primary root phenotype due to the loss of *CEP2* is more likely resulting from the shortened epidermal cells such as trichoblasts. Thus, CEP2 participates in elongating the primary roots. The loss of function mutant phenotype demonstrates that CEP2 activity is required for cell elongation, possibly due to its cell wall remodeling ability. In contrast, since loss of *CEP1* does not result in shortened primary roots, CEP1 does not appear to be involved in primary root elongation.

Pro-CEP2 accumulates within the protoplasm of the longest from the upper end of the LR cap prepared for PCD (PCD site I) at the level of the transition zone and is still present in root cap corpses sticking to epidermis cells in the rapid elongation zone. Furthermore, we used a specific antibody to a peptide in the mature protein of CEP2 which recognizes the mature protein [6]. The CEP2 protein was immunolocalized to root cap corpses sticking to the cell wall of elongating epidermal cells at the beginning of the rapid elongation zone; CEP2 immunolocalization to the apoplast and to LR cap cells below prepared for PCD cannot be excluded. In these cell types, CEP2 could be involved in loosening the neighbouring cell wall for extension. The pro-form of KDEL CysEP is readily converted to the matureted protein at pH below 6.5. In *Arabidopsis*, the apoplastic pH in the LR cap is 5.3–5.5 and in the elongation zone 4.8–4.9, respectively [39]. At this pH, the relative enzymatic activity of the maturated CEP2 can be measured unequivocally within one minute. If CEP2 would be transported or would diffuse to the cell wall as the enzymatically inactive pro-enzyme, it would be immediately transformed by autocatalytic cleavage at a pH below 6.5 to the enzymatically active protein [3, 6]. Our data indicate the possibility that CEP2 activity released from root cap corpses and also from root cap cells underneath prepared for PCD can reach the neighbouring apoplast and cell wall thus impacting epidermal cell elongation.

We demonstrated expression of pro-EGFP-CEP1 and pro-mCherry-CEP2 at stage VII and VIII of LR primordium development in the protoplasm of those root epidermal cells that are separated to enable LR emergence. *CEP1* and *CEP2*, respectively, were expressed only in the two epidermal cells surrounding the emerging lateral root and in the epidermal cells in their direct neighbourhood. Normally, both cells are curved [40]. In some cases, depending on the specific site of the emerging LR, bending of just one epidermal cell seems to be enough for separation thus giving room to the emerging root tip. *CEP1* and *CEP2* exhibit a high degree of fine tuning concerning their gene expression and protein activity in a specific cell depending on the developmental program. Pro-mCherry-CEP2 was also detected at stage I-VI. GUS expression driven from the *CEP1*-promoter was detected at stage I-VI [10], whereas expression of pro-EGFP-CEP1 at stage I-VI might be below detection level of the CLSM used. c*ep1* or *cep2* single loss of function caused the strongest phenotype in retardation of LR emergence, whereas *cep1 cep2* double loss of function did not exhibit an enhanced phenotype. We speculate that in case of total loss of *CEP* the plant might recruit other proteases that are present in the course of lateral root emergence [41].

In angiosperms, LRs are derived from the pericycle layer deep within the parent root tissues [33, 34]. The *Arabidopsis* root has a simple structure composed of the stele surrounded by the three one-cell layers endodermis, cortex and epidermis with the pericycle forming the outermost layer of the stele. The LR primordium penetrates these three cell layers in a spatiotemporally controlled process. The coordination of LR formation and emergence is controlled by auxin, which regulates the properties of cells overlaying LR primordia [42]. *NAC1*, a member of the *NAC* family, is induced by auxin and mediates auxin signaling to promote LR development [43]. Several genes encoding cell wall remodeling enzymes including polygalacturonase (*PG*), xyloglucan:xyloglucosyltransferase (XTR6) and subtilisin-like protease (*AIR3)* are induced by auxin in front of the LR primordium and promote cell separation [44]. By studying *de novo* root organogenesis using leaf explants of *Arabidopsis*, it was shown that wounding not only triggers the auxin-mediated fate transition of regeneration competent cells, but also induces the *NAC* pathway for root tip emergence [45]. The *NAC1* transcription factor was specifically expressed in response to wounding. Here, the *NAC1* pathway functioned independently of auxin and regulated expression of *CEP* genes. *NAC1* overexpression induced many genes during *de novo* root organogenesis, especially *CEP1* and *CEP2*. Up-regulation of *CEP* might be related to the degradation of extensins, which would promote wound healing, and this might be a barrier for regenerated root tip emergence [45].

For the involvement of CEP2 in tissue remodelling involving cell wall extension, the matureted protein should act within the cell wall. The CEP2 immunolocalization appears to be inside cell corpses or even apoplastic. In any case, CEP2 can diffuse out of the corpses into the apoplast of expanding epidermal cells.

It remains to be elucidated how the pro-enzymes of KDEL-CysEP as they are stored in the protoplasm in ER-derived compartments, known as ricinosomes, KDEL-tailed protease-accumulating vesicles (KVs) and ER-bodies [3, 5–7], are transported to the cell wall. A Golgi-dependent secretion pathway is assumed for cell wall remodelling enzymes, such as pectin methylesterases and polygalacturonases (PG), and for xyloglucan-endoglucanases/hydrolases (XTH) involved in cell elongation [46–49]. Since KDEL-CysEP are not glycosylated, as known for their founding member RcCysEP [12] and since they exhibit the ER retention signal [6], a Golgi-independent pathway including membrane fusion is postulated. In germinating mung bean (*Vigna mungo*) cotyledons, their KDEL-CysEP (Sulfhydryl-endopeptidase, SH-EP) is accumulated in KV for mass transport to protein storage vacuoles (PSV) [5]. KV bud from the ER and transport the pro-SH-EP in a Golgi-independent pathway directly to PSV. After fusion of the two membranes, the pro-SH-EP is converted to the mature SH-EP due to the low pH within the PSV and participates in storage protein mobilization [50]. ER bodies are produced by plants of the *Brassicales* order, which includes *Arabidopsis*. Pathogenesis-related proteins (PR), which have antimicrobial activity, accumulated in ER-bodies at a steady-state level and were secreted to the apoplast in response to attack by pathogenic fungi. This suggests that ER bodies are storage sites for various PR proteins released in response to pathogenic attack (for review see [7, 51]). As ricinosomes are storage sites for KDEL-CysEPs, a transport similar to KVs and ER bodies to the apoplast by membrane fusion and/or secretion can be anticipated.

CEP1 is involved in tapetal PCD and pollen development [20]. At stage 5 of tapetal development, CEP1 was concentrated in the ricinosome-like precursor protease vesicles of the tapetal cytoplasm. At early stage 6, CEP1 appeared in tapetal cell vacuoles with fusion of these protease vesicles and transformed into the mature protein before rupture of the vacuole. Subsequently, the CEP1 was released from the vacuoles into the tapetal cytosol. CEP1 was also released to the cell wall by the fusion of small vesicles with the cell membrane during late stage 6 and stage 7. At late stage 7, the CEP1 entered the callose wall and probably participated in callose wall degradation [20]. We infer that KDEL-CysEPs can be transported to the cell wall by fusion of ER-derived vesicles with the plasma membrane. In the apoplast, the pH is acidic enough to activate the accumulated KDEL-CysEP for release to tissues undergoing collapse.

Interestingly, a number of cell wall proteins are upregulated in *cep1* mutants. These include three extensin family genes and other cell wall biogenesis genes such as proline-rich protein 4 (*PRP4*), xyloglucan endotransglycosylase/hydrolase 3 (*XTH3*) and pectin methylesterase 44 (PME44), suggesting that *CEP1* is important for tapetal cell wall organization and degradation [20]. The moderately glycosylated extensins belonging to the family of hydroxyproline-rich glycoproteins are important and abundant structural proteins in the primary cell wall [11, 52–54]. The *Arabidopsis* genome encodes 59 extensin genes. Discovery of the lethal *rsh* (*root-*, *shoot*, *hypocotyl-defective*) embryogenic *Arabidopsis* mutant corresponding to EXT3 showed that extensins are essential for cell plate formation, evidenced by the aberrant mutant wall phenotype and EXT3 immunocytochemical localization [11, 52, 55].

Extensins are ubiquitous in all cell walls including LR cap cells and epidermal as well as cortical cells from the root tip up to the differentiation / root hair zone. The amount of extensins in the respective root cell walls varies considerably. Extensins are found predominantly in epidermal and cortical cell walls of the starting, rapid elongation zone with an increase in cell length up to 300% within less than 3 hours and root upwards to the late elongation zone with cells having already reached their maximal length [32]; comparably small amounts of extensins are in the LR cap cell walls [35]. Remodeling enzymes loosening the rigid cell wall structure are decisive for cell elongation [49, 56, 57]. The development of the *Arabidopsis* LR cap comprises cell elongation [58]. The relationship between the amount of CEP and extensin should influence the degree of cell wall attack. CEP2 is localized in root cap corpses sticking to epidermal cell walls at the beginning rapid elongation zone and in LR cap cells that are elongating before they are shed (PCD site I) and is thus directly involved in cell wall modulation. In a differentiated root, we see CEP signals only in selected epidermis cells supporting the LR outgrowth and within the stele.

Out data set the stage for uncovering novel roles for KDEL-CysEP in cells that are not undergoing PCD but are characterized by extensive cell wall expansion or remodeling possibly due to their cleavage of extensins. The involvement of a protease in cell wall remodeling points to the importance of the cell wall protein moiety in this process. It will be interesting to obtain further insights into the underlying mechanisms such as the membrane fusion of ricinosomes with the plasma membrane in the course of root development similar to other ER-derived vesicles. Upregulation of cell proteins such as extensins is already demonstrated for *cep1* mutants in tapetal cell wall organization. It will be interesting to see whether extensin cleavage and its regulation determines the impact of KDEL-CysEPs, with extensive extensin cleavage resulting in the total breakdown of the cell wall and ensuing cell collapse, while more limited extensin cleavage would enable cell wall remodeling in the context of cell elongation or cell separation.

## Supporting information

S1 Fig Transcripts for *CEP2* in WT and ko mutant. Homozygous knock out mutant plants were obtained for *cep2* (SALK_079519; T-DNA insertion in the second exon). No corresponding transcript could be amplified by RT-PCR using primers that comprise the complete coding region (spanning the T-DNA) from seven days old seedlings, whereas the parent Col-0 wild type expressed the gene. Fw: GATATTTCTCTTTTCTCTTGTCA binding 17bp downstream of the start ATG; rv: CTAGAGCTCATCTTTGACATCACC binding at the stop TAG; Controls: WT, RT-PCR on wild type RNA; WT gen, PCR on genomic DNA; Actin Control, RT-PCR with actin primers. MM, molecular weight markers.
(PDF)

S2 Fig *CEP2* expression in independent lines of *cep1/2* double ko/kd mutants (*cep1*+*cep2-*RNAi). Relative gene expression of *CEP2* was measured in seven days old seedlings. qRT-PCR was performed with *CEP2* gene-specific primers. Expression levels were normalized to the reference gene *ACT8* and expression in wild type Col-0 was set to 1. Error bars represent standard error of the mean (SE). The results were similarly reproduced in a second independent experiment (biological replicate).
(PDF)

S3 Fig Morphological organization of the *Arabidopsis* root tip. **(**Kindly provided by Yvon Jaillais (ENS Lyon; yvon.jaillais@ens-lyon.fr)
(PDF)

S4 Fig *cep2* ko and *cep1/cep2* double ko/kd mutants (*cep1*+*cep2-*RNAi) exhibit a significantly smaller total root width at elongation zone as compared to WT plants. This parallels the trichoblast cell width. (A) Comparison of the root elongation zone width in 7 days old seedlings of WT plants with *cep2* ko and *cep1 cep2* double ko/kd mutants (*cep1*+*cep2-*RNAi; lines 2.21 and 3.14). Columns are marked with different letters indicating statistically different groups for the elongation zone width or with similar letters indicating groups not statistically different according to the ANOVA-and Duncan test. (B) Root elongation zone width expressed as percentage of WT plants. Data represent the respective means of two independent experiments (biological replica) comprising 8 and 9 seedlings per line, respectively.
(PDF)

S5 Fig CEP2 signals are detected within the differentiating protoxylem of the root tip vasculature. The specific CEP2 signals within the differentiating protoxylem indicate that the antibody penetrates all cell layers in the course of whole mount immunolocalization. (A) Overview, root at differentiation zone. VC, vascular cylinder, N, nucleus, E, epidermis. (B) Maximum projection of vascular cylinder; note specific signal accumulation in the stele. S, stele. (C) Optical section with focus plane on vasculature; inset shows CEP2 accumulation around spiral wall thickenings of protoxylem. PX, protoxylem.
(PDF)

S6 Fig Morphological changes during lateral root development. **Lateral roots originate** deep within the primary root from the pericycle cells. The eight stages of primordium development (roman numbers) are shown [adapted from Péret B, Rybel B, de Casimiro I, Benková E, Swarup R, Laplaze L et al. (2009) Arabidopsis lateral root development: an emerging story. Trends in Plant Sci 14: 99–408. doi: 10.1016/j.tplants.2009.05.002].
(PDF)
