# Supplementary material for: The role of KDEL-tailed cysteine endopeptidases of Arabidopsis (AtCEP2 and AtCEP1) in root development
Source: PLoS One. 2018 Dec 21;13(12):e0209407. doi: 10.1371/journal.pone.0209407 (PMC6303060; doi:10.1371/journal.pone.0209407)
Supplement: S1 Fig — Homozygous knock out mutant plants were obtained for cep2 (SALK_079519; T-DNA insertion in the second exon). No corresponding transcript could be amplified by RT-PCR using primers that comprise the complete coding region (spanning the T-DNA) from seven days old seedlings, whereas the parent Col-0 wild type expressed the gene. Fw: GATATTTCTCTTTTCTCTTGTCA binding 17bp downstream of the start ATG; rv: CTAGAGCTCATCTTTGACATCACC binding at the stop TAG; Controls: WT, RT-PCR on wild type RNA; WT gen, PCR on genomic DNA; Actin Control, RT-PCR with actin primers. MM, molecular weight markers. (PDF) [file pone.0209407.s001.pdf]

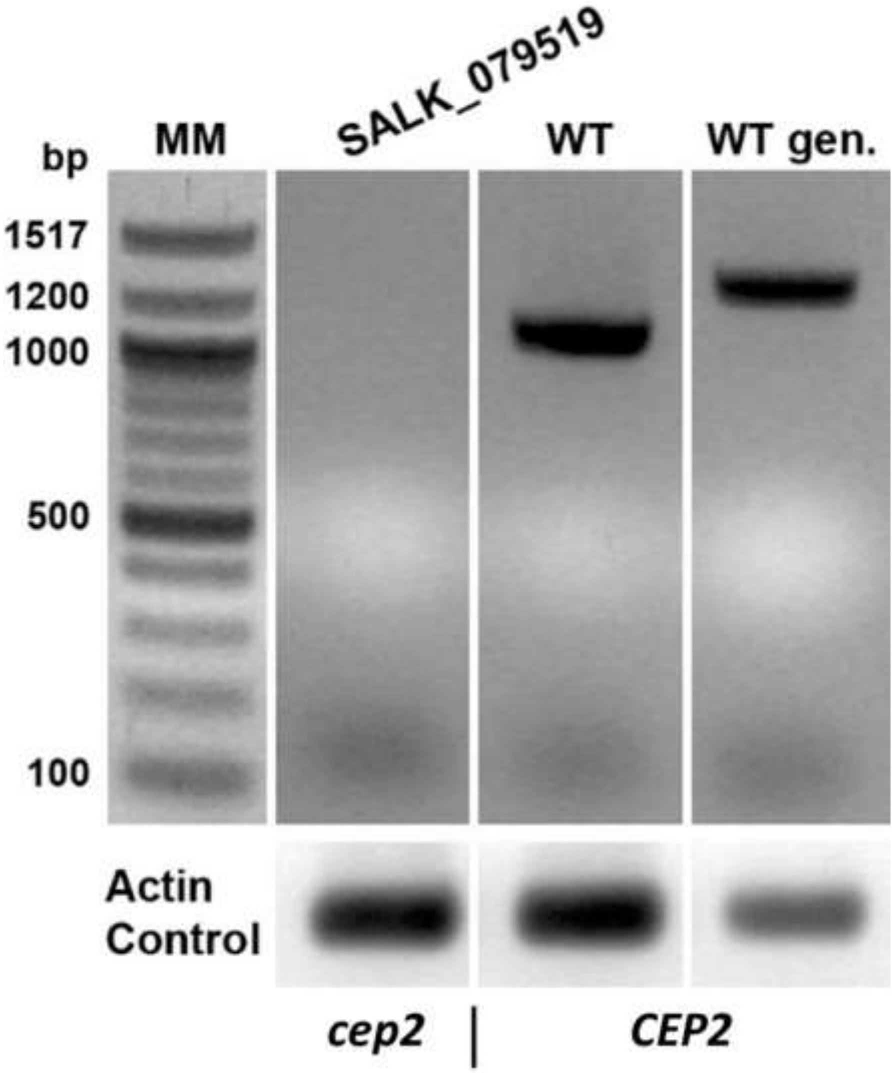

S1 Fig. Transcripts for *CEP2* in WT and ko mutant. Homozygous knock out mutant plants were obtained for *cep2* (SALK_079519; T-DNA insertion in the second exon). No corresponding transcript could be amplified by RT-PCR using primers that comprise the complete coding region (spanning the T-DNA) from seven days old seedlings, whereas the parent Col-0 wild type expressed the gene. Fw: GATATTTCTCTTTTCTCTTGTCA binding 17bp downstream of the start ATG; rv: CTAGAGCTCATCTTTGACATCACC binding at the stop TAG; Controls: WT, RT-PCR on wild type RNA; WT gen, PCR on genomic DNA; Actin Control, RT-PCR with actin primers. MM, molecular weight markers.
