# Supplementary material for: The role of KDEL-tailed cysteine endopeptidases of Arabidopsis (AtCEP2 and AtCEP1) in root development
Source: PLoS One. 2018 Dec 21;13(12):e0209407. doi: 10.1371/journal.pone.0209407 (PMC6303060; doi:10.1371/journal.pone.0209407)
Supplement: S2 Fig — Relative gene expression of CEP2 was measured in seven days old seedlings. qRT-PCR was performed with CEP2 gene-specific primers. Expression levels were normalized to the reference gene ACT8 and expression in wild type Col-0 was set to 1. Error bars represent standard error of the mean (SE). The results were similarly reproduced in a second independent experiment (biological replicate). (PDF) [file pone.0209407.s002.pdf]

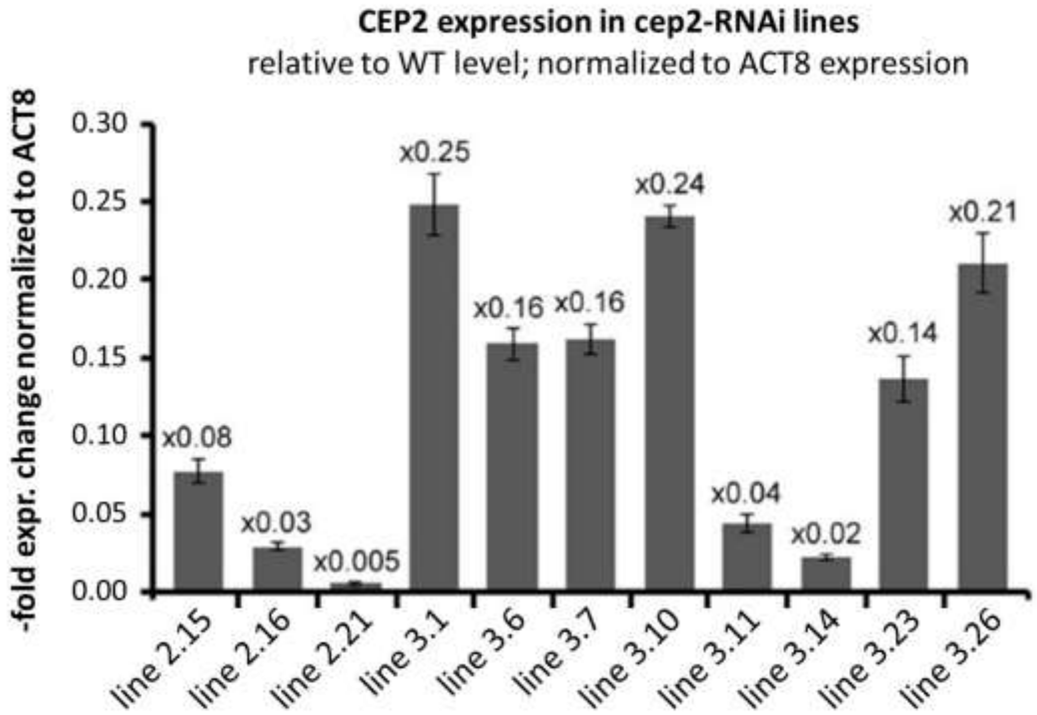

S2 Fig. *CEP2* expression in independent lines of *cep1/2* double ko/kd mutants (*cep1+cep2*-RNAi). Relative gene expression of *CEP2* was measured in seven days old seedlings. qRT-PCR was performed with *CEP2* gene-specific primers. Expression levels were normalized to the reference gene *ACT8* and expression in wild type Col-0 was set to 1. Error bars represent standard error of the mean (SE). The results were similarly reproduced in a second independent experiment (biological replicate).
