# Supplementary material for: The role of KDEL-tailed cysteine endopeptidases of Arabidopsis (AtCEP2 and AtCEP1) in root development
Source: PLoS One. 2018 Dec 21;13(12):e0209407. doi: 10.1371/journal.pone.0209407 (PMC6303060; doi:10.1371/journal.pone.0209407)
Supplement: S3 Fig — (Kindly provided by Yvon Jaillais (ENS Lyon; yvon.jaillais@ens-lyon.fr) (PDF) [file pone.0209407.s003.pdf]

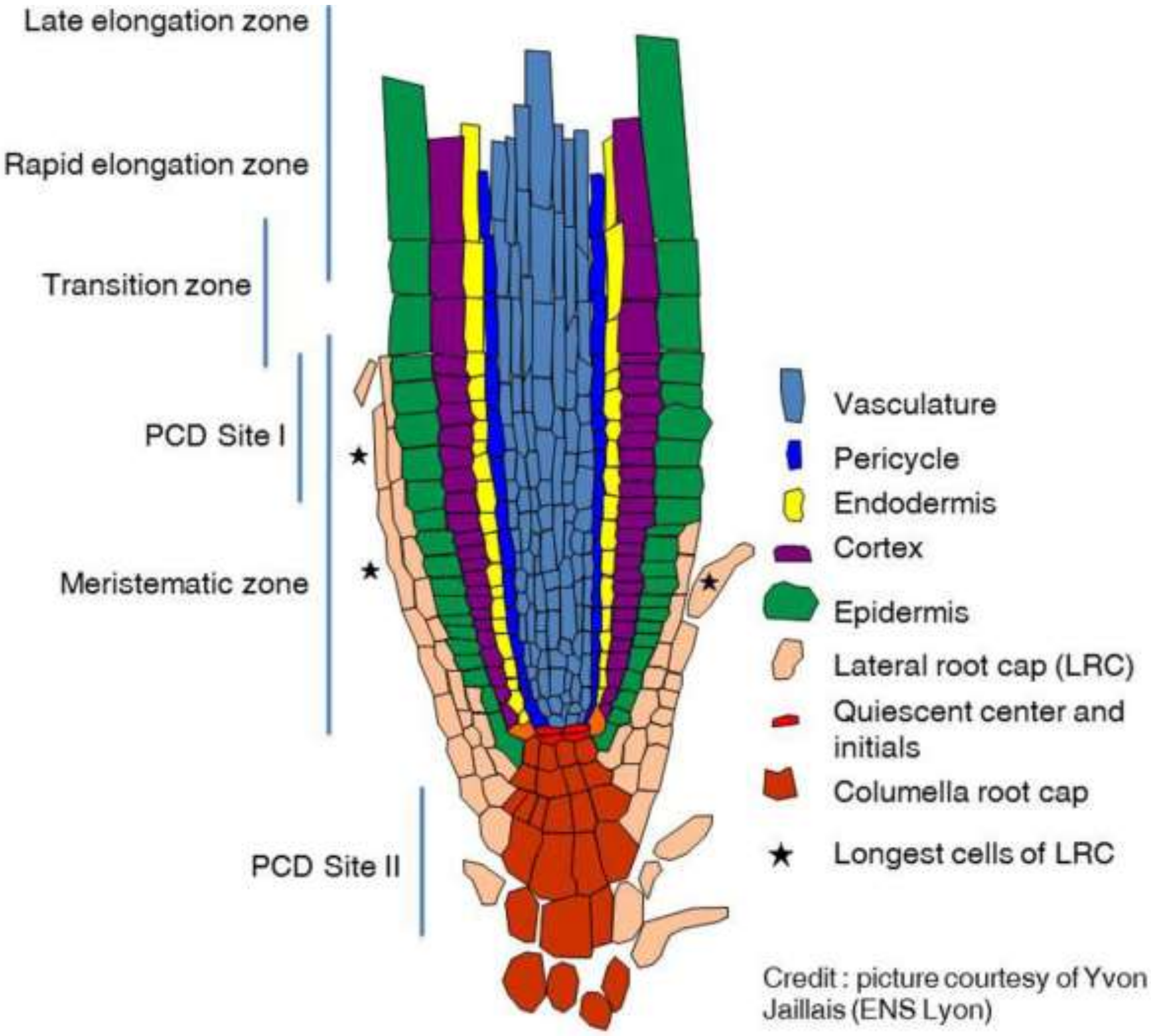

S3 Fig. Morphological organization of the *Arabidopsis* root tip. (Kindly provided by Yvon Jaillais (ENS Lyon; yvon.jaillais@ens-lyon.fr)
