# Supplementary material for: The role of KDEL-tailed cysteine endopeptidases of Arabidopsis (AtCEP2 and AtCEP1) in root development
Source: PLoS One. 2018 Dec 21;13(12):e0209407. doi: 10.1371/journal.pone.0209407 (PMC6303060; doi:10.1371/journal.pone.0209407)
Supplement: S4 Fig — This parallels the trichoblast cell width. (A) Comparison of the root elongation zone width in 7 days old seedlings of WT plants with cep2 ko and cep1 cep2 double ko/kd mutants (cep1+cep2-RNAi; lines 2.21 and 3.14). Columns are marked with different letters indicating statistically different groups for the elongation zone width or with similar letters indicating groups not statistically different according to the ANOVA-and Duncan test. (B) Root elongation zone width expressed as percentage of WT plants. Data represent the respective means of two independent experiments (biological replica) comprising 8 and 9 seedlings per line, respectively. (PDF) [file pone.0209407.s004.pdf]

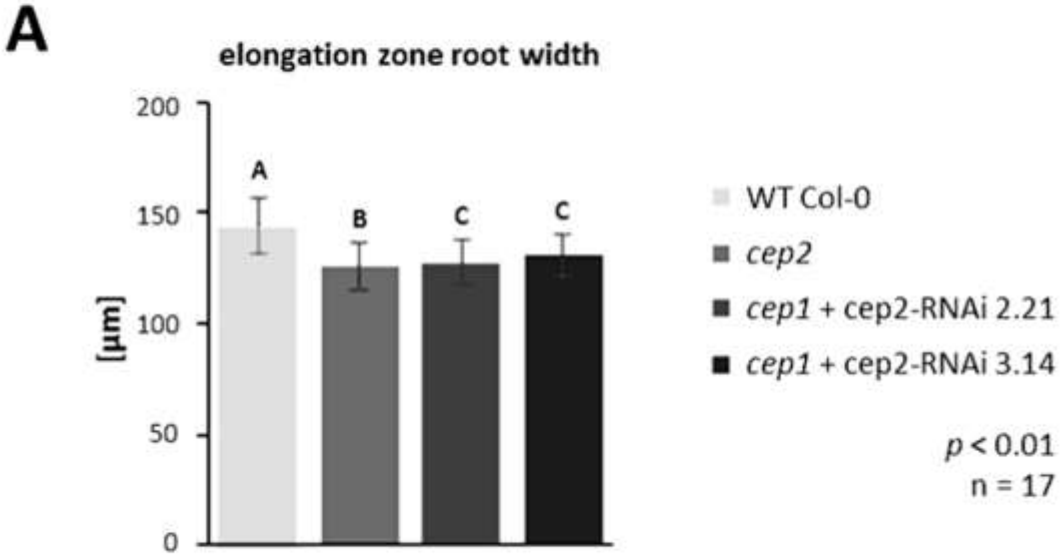

B

	trichoblast cell width relative to WT
cep2	87 %
cep1 + cep2-RNAi 2.21	89 %
cep1 + cep2-RNAi 3.14	90 %

S4 Fig. *cep2* ko and *cep1/cep2* double ko/kd mutants (*cep1+cep2*-RNAi) exhibit a significantly smaller total root width at elongation zone as compared to WT plants. This parallels the trichoblast cell width. (A) Comparison of the root elongation zone width in 7 days old seedlings of WT plants with *cep2* ko and *cep1 cep2* double ko/kd mutants (*cep1+cep2*-RNAi; lines 2.21 and 3.14). Columns are marked with different letters indicating statistically different groups for the elongation zone width or with similar letters indicating groups not statistically different according to the ANOVA-and Duncan test. (B) Root elongation zone width expressed as percentage of WT plants. Data represent the respective means of two independent experiments (biological replica) comprising 8 and 9 seedlings per line, respectively.
