# Supplementary material for: The role of KDEL-tailed cysteine endopeptidases of Arabidopsis (AtCEP2 and AtCEP1) in root development
Source: PLoS One. 2018 Dec 21;13(12):e0209407. doi: 10.1371/journal.pone.0209407 (PMC6303060; doi:10.1371/journal.pone.0209407)
Supplement: S5 Fig — The specific CEP2 signals within the differentiating protoxylem indicate that the antibody penetrates all cell layers in the course of whole mount immunolocalization. (A) Overview, root at differentiation zone. VC, vascular cylinder, N, nucleus, E, epidermis. (B) Maximum projection of vascular cylinder; note specific signal accumulation in the stele. S, stele. (C) Optical section with focus plane on vasculature; inset shows CEP2 accumulation around spiral wall thickenings of protoxylem. PX, protoxylem. (PDF) [file pone.0209407.s005.pdf]

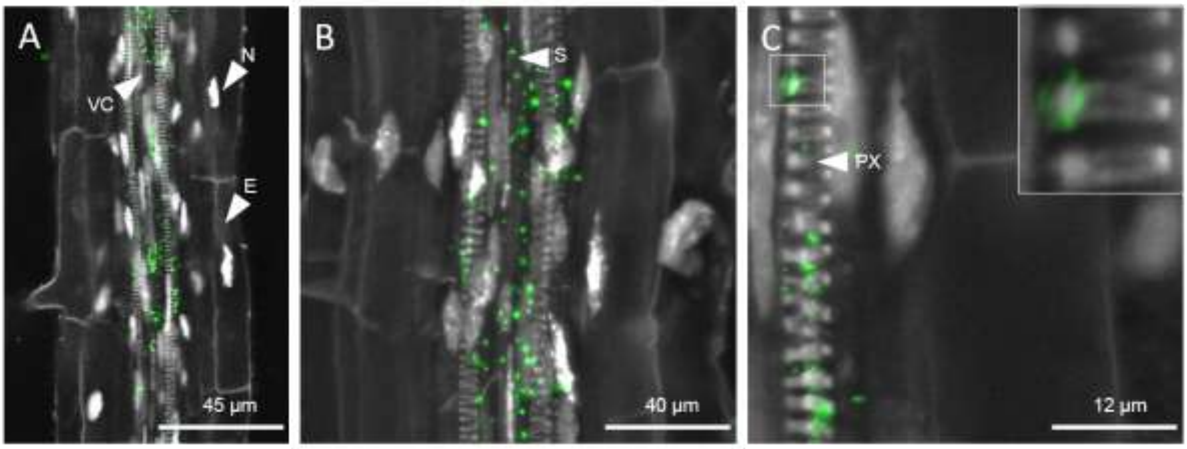


**S5 Fig. CEP2 signals are detected within the differentiating protoxylem of the root tip vasculature.** The specific CEP2 signals within the differentiating protoxylem indicate that the antibody penetrates all cell layers in the course of whole mount immunolocalization. (A) Overview, root at differentiation zone. VC, vascular cylinder, N, nucleus, E, epidermis. (B) Maximum projection of vascular cylinder; note specific signal accumulation in the stele. S, stele. (C) Optical section with focus plane on vasculature; inset shows CEP2 accumulation around spiral wall thickenings of protoxylem. PX, protoxylem.
