# Supplementary material for: The role of KDEL-tailed cysteine endopeptidases of Arabidopsis (AtCEP2 and AtCEP1) in root development
Source: PLoS One. 2018 Dec 21;13(12):e0209407. doi: 10.1371/journal.pone.0209407 (PMC6303060; doi:10.1371/journal.pone.0209407)
Supplement: S6 Fig — Lateral roots originate deep within the primary root from the pericycle cells. The eight stages of primordium development (roman numbers) are shown [adapted from Péret B, Rybel B, de Casimiro I, Benková E, Swarup R, Laplaze L et al. (2009) Arabidopsis lateral root development: an emerging story. Trends in Plant Sci 14: 99–408. doi: 10.1016/j.tplants.2009.05.002]. (PDF) [file pone.0209407.s006.pdf]

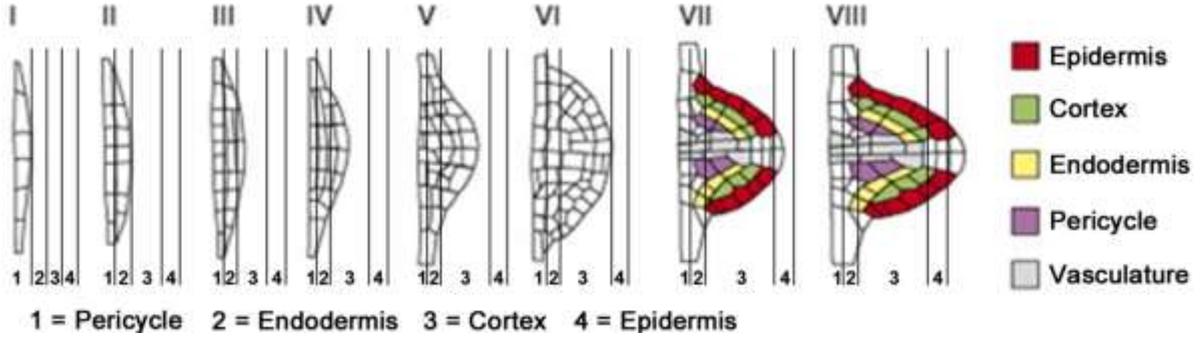

S6 Fig. Morphological changes during lateral root development. Lateral roots originate deep within the primary root from the pericycle cells. The eight stages of primordium development (roman numbers) are shown [adapted from Péret B, Rybel B, de Casimiro I, Benková E, Swarup R, Laplaze L et al. (2009) Arabidopsis lateral root development: an emerging story. Trends in Plant Sci 14: 99–408. doi:10.1016/j.tplants.2009.05.002].
